# A serine protease secreted from *Bacillus subtilis* cleaves human plasma transthyretin to generate an amyloidogenic fragment

**DOI:** 10.1038/s42003-020-01493-0

**Published:** 2020-12-11

**Authors:** Daniele Peterle, Giulia Pontarollo, Stefano Spada, Paola Brun, Luana Palazzi, Alexej V. Sokolov, Barbara Spolaore, Patrizia Polverino de Laureto, Vadim B. Vasilyev, Ignazio Castagliuolo, Vincenzo De Filippis

**Affiliations:** 1 Department of Pharmaceutical and Pharmacological Sciences, University of Padua, Padua, Italy; 2 Department of Molecular Medicine, University of Padua, Padua, Italy; 3 Institute for Experimental Medicine, Saint Petersburg, Russia; 4 Saint Petersburg University, Saint Petersburg, Russia; 5 CRIBI Biotechnology Center, University of Padua, Padua, Italy; 6 Present Address: Department of Chemistry and Chemical Biology, Northeastern University, 360 Huntington Ave., Boston, MA 02115 USA; 7 Present Address: Center for Thrombosis and Hemostasis (CTH) University Medical Center Mainz, Langenbeckstraße 1, 55131 Mainz, Germany

**Keywords:** Proteases, Risk factors

## Abstract

Aggregation of human wild-type transthyretin (hTTR), a homo-tetrameric plasma protein, leads to acquired senile systemic amyloidosis (SSA), recently recognised as a major cause of cardiomyopathies in 1–3% older adults. Fragmented hTTR is the standard composition of amyloid deposits in SSA, but the protease(s) responsible for amyloidogenic fragments generation in vivo is(are) still elusive. Here, we show that subtilisin secreted from *Bacillus subtilis*, a gut microbiota commensal bacterium, translocates across a simulated intestinal epithelium and cleaves hTTR both in solution and human plasma, generating the amyloidogenic fragment hTTR(59–127), which is also found in SSA amyloids in vivo. To the best of our knowledge, these findings highlight a novel pathogenic mechanism for SSA whereby increased permeability of the gut mucosa, as often occurs in elderly people, allows subtilisin (and perhaps other yet unidentified bacterial proteases) to reach the bloodstream and trigger generation of hTTR fragments, acting as seeding nuclei for preferential amyloid fibrils deposition in the heart.

## Abstract (web summary)

Peterle et al. show that a subtilisin like serine protease secreted from gut microbiota *Bacillus subtilis* cleaves the wild-type human transthyretin (hTTR) to generate an amyloidogenic peptide. High propensity of the hTTR fragment to form pathogenic protein aggregates implicates the serine protease in the pathogenesis of acquired senile systemic amyloidosis.

## Introduction

Human transthyretin (hTTR) is a 55-kDa homotetrameric protein, mainly produced in the liver and brain, and abundantly present in human plasma (0.18–0.45 mg/ml)^1^. In its native tetrameric conformation, hTTR transports thyroid hormones in the blood and cerebrospinal fluid along with retinol as a binding partner of retinol binding protein^1^. Each hTTR monomer consists of 127 amino acids and forms eight β-strands, named from A to H, which are arranged in a β-sandwich of two β-sheets (I and II) and one small α-helix. Each β-sheet is formed by four strands (β-sheet I: H-G-A-D strands; β-sheet II: C-B-E-F strands), while the α-helix is located between strands E and F^2–4^. The monomers tightly interact, mainly through hydrogen bonds networks between adjacent antiparallel β-strands H-H′ and F-F′, to form stable dimers. The two dimers then weakly interact to form the final tetrameric structure, stabilized by hydrophobic interactions involving the A-B and G-H loops^2–4^.

hTTR is one of the 25 proteins related to the onset of amyloid-based diseases^5^ and, indeed, hTTR aggregation causes amyloidosis, which is associated to two different pathological conditions, i.e., familial amyloidosis (ATTR) and senile systemic amyloidosis (SSA)^6^. ATTR is a rare hereditary disorder with a relatively early age of onset, usually <60 years, characterized by the deposition of hTTR amyloid fibrils in different organs and generally leading to polyneuropathy and/or cardiomyopathy^7,8^. ATTR has been associated with >100 single point mutations affecting the hTTR gene and it has been recognized as the most common cause of hereditary amyloidosis worldwide^9^. Nevertheless, the prevalence of ATTR in Europe is <1 in 100,000 and only about 10,000 cases of ATTR have been estimated in the global population^7,8^. At variance with ATTR, SSA involves wild type hTTR, more often affects elderly people (usually >75 years), it is generally associated to cardiomyopathic complications, and leads to progressive heart failure^10,11^. Recent data suggest that SSA-related cardiomyopathy has been often overlooked as a common cause of heart failure in older adults^12–18^. Indeed, hTTR amyloid fibrils were found in 25% of post-mortem hearts from patients >80 years of age^11^. Furthermore, the use of non-invasive imaging techniques recently allowed to reveal the presence of hTTR amyloid deposits in about 15% of older patients with heart failure or aortic stenosis and to estimate a prevalence of hTTR-related cardiomyopathy in 1–3% of elderly people >75 years of age^17,18^.

Analysis of hTTR amyloid deposits that are found in vivo allowed to identify two distinct types of amyloid fibrils, i.e., type A and type B fibrils. Type A fibrils are short-sized and mainly contain C-terminal fragments of hTTR with some intact hTTR molecules. At variance, type B fibrils have an elongated/slender shape and almost exclusively contain full-length hTTR^19,20^. Mounting evidences accumulated in the last two decades indicate that fragmented hTTR, represented by a mixture of C-terminal fragments starting at positions from 44 to 59, is the standard composition of amyloid deposits of wild type and mutant hTTR in patients with either hereditary ATTR^9,19–23^ or acquired SSA^19,24,25^. However, the fibril types are correlated to phenotypic differences, as patients with a relative abundance of C-terminal fragments (i.e., type A fibrils) have a late onset and develop cardiomyopathy, while patients containing more intact hTTR (i.e., type B fibrils) in their amyloid deposits have an early onset and less myocardial involvement^9,19–25^. Although the protease(s) responsible for proteolysis-induced amyloidosis by hTTR in vivo has(ve) not yet been certainly identified, recent findings by Bellotti and co-workers indicate that digestive (i.e., trypsin)^9^ and fibrinolytic (i.e., plasmin)^26^ proteases cleave hTTR, under shear stress conditions in vitro, and generate the amyloidogenic fragment hTTR(49–127), which has been also found in both SSA and ATTR amyloid deposits^9,19–25^.

Whereas the pathogenesis of genetic ATTR can be rationalized considering the effects that amino acid mutations have on hTTR tetramer structure, the cause of the late onset SSA in elderly people is much more difficult to decipher. For ATTR, in fact, it is widely accepted that amyloidogenic point mutations destabilize hTTR tetrameric structure, shifting the tetramer ↔ dimer ↔ monomer equilibrium towards the monomeric form, which is more prone to form amyloid fibrils and/or to be susceptible to proteolysis, generating amyloidogenic fragments^26–28^. In the case of SSA, instead, the molecular events leading to the late onset of cardiac hTTR amyloid deposits in elderly patients remains mysterious, despite normal hTTR circulating in their blood from birth^26^. All these considerations suggest that non-genetic, age-related factors must play a role in the pathogenesis of SSA.

Hence, we decided to screen several different intestinal, coagulative, fibrinolytic, leukocyte, and bacterial proteases with respect to their ability to cleave wild-type natural plasma hTTR and generate amyloidogenic fragments. Among the tested proteases, only subtilisin, a serine protease secreted from *Bacillus subtilis* (a non-pathogenic component of the normal gut microbiota), can trigger hTTR amyloid deposition by efficiently cleaving purified hTTR at the Leu58-Thr59 bond, either under static (pH 7.4, 37 °C) and shear stress conditions in vitro. The resulting C-terminal fragment hTTR(59–127) is resistant to further proteolysis and forms typical amyloid fibrils, as detected by thioflavin T (ThT) binding assay and transmission electron microscopy (TEM) analysis. We have also demonstrated that subtilisin can efficiently translocate across a carcinoma colon-2 (CaCo-2) cells simulated intestinal epithelium, and generate the amyloidogenic fragment 59–127 in human plasma. Noteworthy, the same fragment hTTR(59–127) was found in the amyloid deposits of patients with hTTR amyloidosis^22,23^.

## Results

### Identification of subtilisin as a hTTR-cleaving protease

hTTR was first purified to homogeneity from human plasma by the phenol precipitation method^29^, followed by ion-exchange chromatography and size-exclusion chromatography (Supplementary Fig. 1), allowing to obtain in good yields (>35%) highly pure (>98%) hTTR preparations, as obtained by polyacrylamide gel electrophoresis in the presence of sodium dodecyl sulfate (SDS-PAGE), where hTTR predominantly migrates as a monomer at ~13 kDa while minute amounts of hTTR dimer are still present in the denaturing sample loading buffer of the SDS-PAGE (Supplementary Fig. 1C), as already reported^30^. In keeping with literature data^31^, RP-HPLC and high-resolution MS analyses show extensive chemical modification of purified hTTR at Cys-10, whereby the S^λ^-cysteinyl derivative (S-Cys-hTTR) is the most abundant isoform (52 ± 6%). Circular dichroism and fluorescence spectra, along with analytical size-exclusion chromatography and dynamic light scattering (DLS) measurements indicate that our purified hTTR has conformational properties identical to those of other plasma hTTR preparations^32^ and that it exists as a mono-dispersed tetramer, with an apparent molecular weight of 57 ± 3 kDa and a hydrodynamic radius (*R*_h_) of 3.8 ± 1.0 nm, very close to that determined earlier^33^ (Supplementary Fig. 2 and Table 1).

**Fig. 1:**
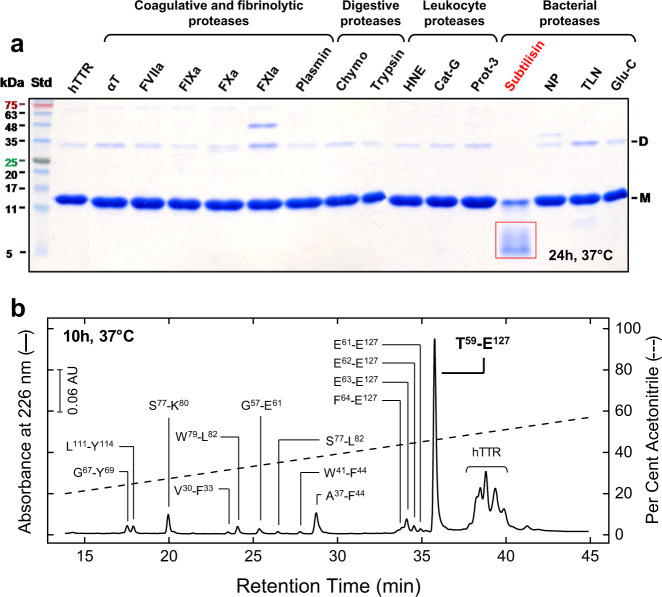
Proteolysis of hTTR by different proteases. **a** hTTR (1 mg/ml) was reacted at 37 °C in TBS pH 7.4, containing 5 mM CaCl_2_, with different proteases at an enzyme:hTTR ratio of 1:20 (mol/mol): trypsin and chymotrypsin, α-thrombin (αT), activated factors VII, IX, X, and XI, plasmin, human neutrophil elastase (HNE), cathepsin-G (Cat-G) and Proteinase-3 (Prot-3), subtilisin and neutral protease (NP) from *Bacillus subtilis*, thermolysin (TLN) from *Bacillus thermoproteolyticus*, and Glu-C specific endoproteinase (Glu-C) from *Staphylococcus aureus*. After 24-h reaction, the proteolysis mixtures were analysed by reducing SDS-PAGE (4–15% acrylamide) and Coomassie stained. hTTR was resistant to all proteases tested, except to subtilisin (red). Gels bands corresponding to the monomeric (M) and dimeric form (D) of hTTR are indicated. Shallow bands at molecular weights higher than hTTR dimers are assigned to the proteases added. **b** RP-HPLC analysis of the proteolysis reaction of hTTR with subtilisin. The reaction was conducted as in **a**. After 10-h reaction, the proteolysis mixture was acid-quenched and fractionated on an analytical C18 column. Fractions were collected and analysed by high-resolution MS, allowing to establish the chemical identity of the peptide material eluted in correspondence of the chromatographic peaks. Small fragments were identified in most cases. Of note, the fragment eluting with the major chromatographic peak was identified as Thr59-Glu127 peptide (7757.4 Da) and was found to be resistant to further proteolysis.

Thereafter, purified hTTR was challenged with numerous proteases displaying different substrate specificity and mechanism of action, including digestive, coagulative and fibrinolytic, neutrophil, and bacterial proteases. In all cases, purified hTTR (1 mg/ml) was incubated under static conditions for 24 h at 37 °C in 5 mM Tris-HCl buffer, pH 7.4, 0.15 M NaCl, 5 mM CaCl_2_ (TBS-CaCl_2_) using a protease:hTTR ratio of 1:20 (mol:mol). Proteolysis reactions were analysed by denaturing and native PAGE. SDS-PAGE (Fig. 1a) shows that, among the 15 proteases tested, only *B. subtilis* subtilisin proved to efficiently cleave hTTR. Notably, the lane corresponding to subtilisin-induced proteolysis shows that the intensity of hTTR monomer (M) band strongly decreased along with that of the dimer (D), which was not detectable, whereas a diffused electrophoretic band appeared in the 5–9 kDa range. Besides subtilisin, only in the case of thermolysin a faint band at ~9 kDa was barely detectable while all other proteases failed to cleave hTTR. At variance with SDS-PAGE, the corresponding native PAGE analysis of the proteolysis reaction of hTTR with subtilisin was not very informative, as the newly generated fragment and the residual parent protein co-migrated in a single/diffused band (Supplementary Fig. 3). Indeed, despite the different molecular weight (MW) and isoelectric point (pI) of hTTR(59–127) [MW = 7757 Da, pI = 4.95] and hTTR [MW = 13,761 Da, pI = 5.31], they accidentally share a similar charge/size ratio and thus similar electrophoretic mobility. Subtilisin is a serine protease with very broad substrate specificity, ranging from aromatic to basic and even acidic amino acids, with some preference for large uncharged residues at the primary specificity site^34^. Liquid chromatography mass spectrometry (LC-MS) analysis allowed us to identify hTTR(59–127) as the major proteolytic fragment, resulting from cleavage at the Leu58-Thr59 peptide bond (Fig. 1b). The C-terminal hTTR(59–127) fragment was resistant to further proteolysis, whereas the N-terminal region 1–58 underwent extensive proteolytic degradation at multiple sites, generating very small fragments that allowed us to cover the complete hTTR amino acid sequence (Supplementary Table 2).

**Fig. 2:**
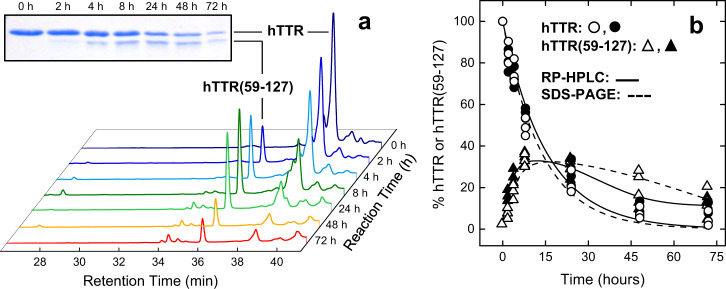
Time-course analysis of hTTR proteolysis by subtilisin. hTTR (1 mg/ml) was reacted in TBS, pH 7.4, containing 5 mM CaCl_2_, with subtilisin (0.9 μM) at an enzyme/hTTR ratio of 1:20 (mol/mol) at 37 °C. At increasing time points, the proteolysis mixtures were acid-quenched and aliquots (20 μg) analysed by RP-HPLC and SDS-PAGE. **a** RP-HPLC waterfall plot of hTTR proteolysis by subtilisin. Aliquots of the reaction mixture were loaded onto a C18 analytical column and eluted with a linear aqueous acetonitrile-0.1% TFA gradient from 10 to 45% in 30 min, at a flow rate of 0.8 ml/min. The absorbance of the effluent was recorded at 226 nm. **Inset** SDS-PAGE (4–20% acrylamide) analysis was carried by loading aliquots (5 μg) of the reaction mixture under reducing conditions, followed by Coomassie staining. **b** Time-course plot of hTTR degradation (circles) and hTTR(59–127) generation (triangles) monitored by RP-HPLC (solid line) or SDS-PAGE (dashed line). Data points were obtained by integrating the chromatographic peak areas or by densitometric analysis of the gel bands in **a**. The data relative to hTTR proteolysis were fitted with Eq. (1) (Supplementary Information), to yield an observed, cumulative kinetic constant, *k*, of hTTR hydrolysis (*k* = 1.6 ± 0.2 × 10^−5^ s^−1^) for both chromatographic and electrophoretic data. The data were obtained from three different measurements. The fitting curves relative to hTTR(59–127) generation are only intended to help the reader to follow the data points.

The time-course kinetics of hTTR proteolysis by subtilisin was monitored by RP-HPLC and SDS-PAGE (Fig. 2). Notably, at reaction times longer than 8 h, the decrease of intact hTTR was not quantitatively compensated by a concomitant increase of hTTR(59–127), likely because the fragment started precipitating from the proteolysis mixture as an insoluble aggregate that could not be detected. With the aim to identify the very first cleavage site in hTTR, the kinetics of proteolysis was also conducted at a ten-fold lower subtilisin:hTTR molar ratio (1:200) (Supplementary Fig. 4). As expected, hTTR(59–127) generation was slower, but still the N-terminal region (1–58) was suddenly proteolyzed at multiple sites, even at short reaction times (5–10 min), impairing identification of a sequential cleavage pattern. Similar results were obtained with the amyloid deposits in both ATTR and SSA patients where, besides C-terminal fragments, only small amounts of short N-terminal fragments could be identified^20,24,25^.

### Probing hTTR dynamics by hydrogen-deuterium exchange mass spectrometry

Protein conformational flexibility is the most important structural property dictating the susceptibility of a given protein site to proteolytic attack, allowing the cleavable segment to adapt to the protease active site with minimal (if any) energetic cost^35^. In the last two decades, hydrogen-deuterium exchange mass spectrometry (HDX-MS) has emerged as a powerful analytical method for investigating protein conformation and dynamics, as backbone amide hydrogens at exposed/flexible sites will exchange rapidly with deuterium, whereas those hydrogens that are buried in the protein interior and involved in bonding interactions will exchange much more slowly^36,37^. HDX can be monitored by recording the time-dependent mass increase of the whole protein (i.e., global exchange analysis) or of short fragments, generated after proteolysis with pepsin (i.e., local exchange analysis). The latter method yields the percentage deuterium uptake (%D) of each peptic fragment and allows to study protein dynamics with a spatial resolution of 3–6 amino acids^36,37^.

**Fig. 3:**
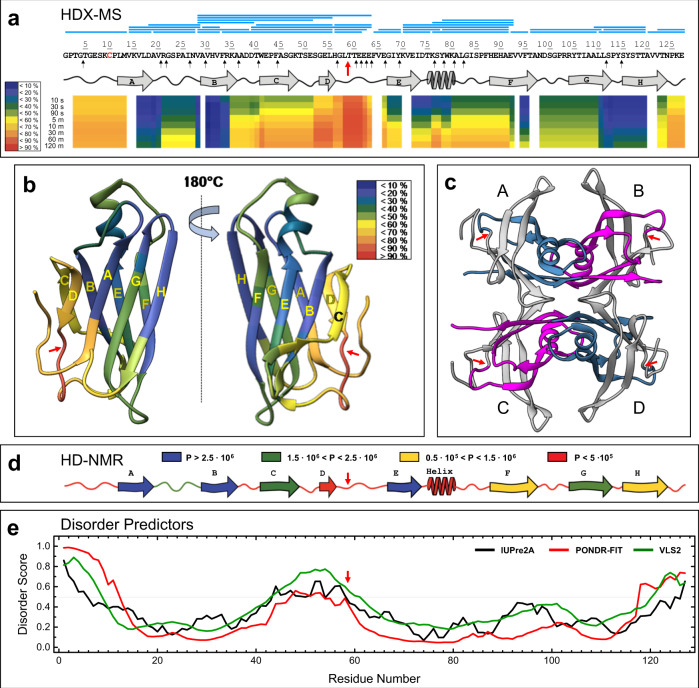
HDX-MS analysis of plasma purified hTTR. **a** Bidimensional HDX-MS heatmap of plasma purified hTTR at pH 7.2. For each peptic fragment (blue bars), the %*D* value at the indicated labeling time (0.5–120 min) is mapped onto the protein sequence. The color key in the heatmap indicates %*D*, from dark blue (<10%) to light red (>90%). Secondary structure is noted at the bottom of hTTR sequence, along with a thick red arrow indicating the major scissile bond (Leu58-Thr59) for subtilisin and small black arrows indicating minor cleavage sites. The heatmap was generated using 38 single and overlapping fragments (Supplementary Fig. 5). **b** Three-dimensional HDX-MS heatmap of deuterium uptake by hTTR was obtained by mapping %*D* values reported in **a** (30-s H/D exchange time) onto the crystal structure of hTTR (1tta.pdb)^2^. For clarity, only the monomer structure is shown. Labels (yellow) identify β-strands while the arrows (red) indicate the scissile Leu58Thr59 bond. **c** Ribbon drawing of hTTR tetramer structure (1tta). The region 59–127 in the interacting (symmetry related) monomers are shown in blue (A, D) and magenta (B, C). The region 1–58 is shown in gray. **d** Schematic representation of HDX-NMR protection factors (P) of recombinant wild-type hTTR, after 2-h exchange time at pH 5.7^38^. The color key indicates *P* values: from blue (*P* > 2.5 × 10^6^) to red (*P* < 0.5 × 10^6^). Flexible/exposed and rigid/buried regions are characterized by low and high *P* values, respectively. **e** Protein disorder profiles of hTTR sequence, according to different prediction methods: IUPre2A^75^, PONDR-FIT^76^, and VSL2^77^.

Here, we carried out a local HDX-MS analysis of purified hTTR at pH 7.2. The bi-dimensional and three-dimensional heatmap representations of %*D* values (Fig. 3a, b) show that even at short exchange times (*t* < 90 s) the D-E loop (Gly57-Glu66) is by far the most flexible segment (%*D* > 90%) of hTTR structure in solution (Supplementary Fig. 5), fully consistent with the preferential subtilisin cleavage occurring at the Leu58-Thr59 bond. The N-terminal segment, part of β-strand *A*, the short β-strand *D*, and the protein C-terminal end are highly flexible (50% < %*D* < 70%), whereas regions encompassing parts of β-strands *A-B*, *E-F*, and *G-H*, along with the short α-helix, are more rigid (10% < %*D* < 40%). Although there is a general increase of %*D* values at longer exchange times, part of β-strands A and F and β-strands B, G and H remain largely protected even after 2-h incubation with D_2_O, consistent with the crystallographic hTTR tetramer, where they are shielded from the solvent and tightly packed in the tetrameric hTTR structure (Fig. 3c). Our HDX-MS data are in general agreement with the results of HDX-nuclear magnetic resonance (NMR) measurements, obtained with recombinant hTTR at pH 5.7, showing that after 2-h exchange the D-E loop, along with the N-terminal and C-terminal segments, is highly flexible and characterized by very low protection factor (*P*) values (Fig. 3d)^38^. Both HDX-MS and HDX-NMR data are in keeping with the theoretical protein disorder profiles of hTTR sequence (Fig. 3e) concurrently predicting that, in addition to the N-terminal and C-terminal regions, hTTR contains a segment (residues 43–61) with high intrinsic disorder probability.

On these grounds, it is conceivable to propose that subtilisin first cleaves the peptide bond Leu58-Thr59 in the highest flexible hTTR region, i.e., the D-E loop. Protease nicking preferentially destabilizes the N-terminal fragment 1-58, lacking strong inter-subunit contacts, which becomes looser and further attacked by the protease. Conversely, the ensuing C-terminal fragment 59–127 is more stable to subtilisin, as it is protected by extensive interactions between the adjacent antiparallel β-strands H-H′ and F-F′ at the dimer interface (Fig. 3d).

### Amyloid fibrils generation during proteolysis of isolated hTTR by subtilisin

**Fig. 4:**
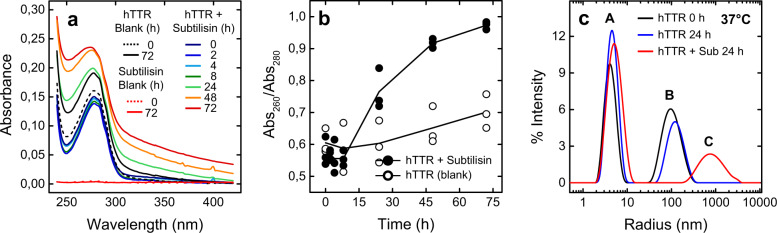
Time-course analysis of hTTR aggregation induced by subtilisin. hTTR (1 mg/ml) was treated at 37 °C with subtilisin in TBS buffer, pH 7.4, containing 5 mM CaCl_2_ at an enzyme:hTTR ratio of 1:20 (mol/mol). At time points, aliquots (50 µl) of the proteolysis reaction were withdrawn, diluted ten-fold with the same buffer and analysed by UV–Vis absorption spectroscopy and DLS. **a** UV–Vis absorption spectra of the proteolysis reaction of hTTR with subtilisin at increasing reaction time. Blank measurements with hTTR and subtilisin alone are also shown. **b** Plot of the Abs_260_/Abs_280_ ratio vs. time of a hTTR solution (0.1 mg/ml) in the presence (black circles) and absence (white circles) of subtilisin. The data points result from three different experiments. **c** DLS analysis of hTTR proteolysis by subtilisin after 24-h reaction. DLS traces of hTTR alone, after 0 or 24-h incubation are shown as controls. DLS data are reported as % intensity size distribution. For each trace, the values of *R*_h_, %mass distribution and %PD of the oligomeric/polymeric species A, B, and C are reported in the Supplementary Table 1.

**Fig. 5:**
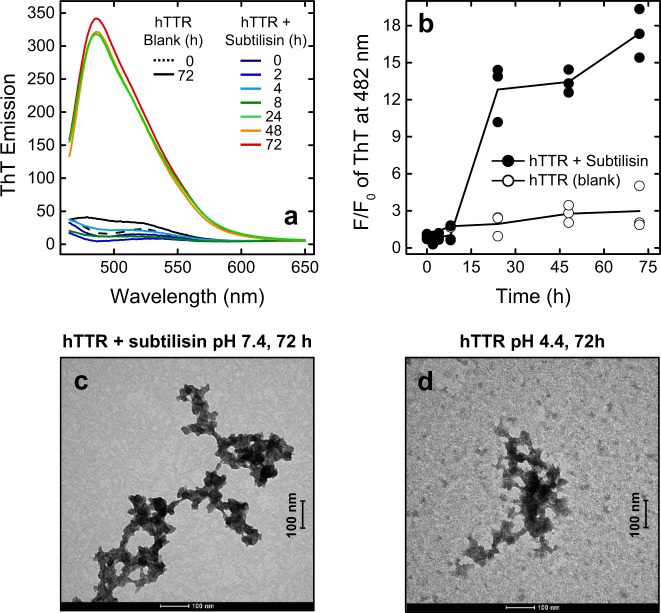
hTTR amyloid fibril formation during proteolysis with subtilisin. hTTR (1 mg/ml) was treated at 37 °C with subtilisin in TBS buffer, pH 7.4, 5 mM CaCl_2_ at an enzyme:hTTR ratio of 1:20 (mol/mol). At time points, aliquots (50 µl) of the proteolysis reaction were withdrawn, diluted ten-fold with the same buffer, and analysed by ThT binding assay and TEM. **a** Fluorescence emission spectra of ThT during proteolysis of hTTR (1 mg/ml) with subtilisin. At the indicated time points, aliquots of the proteolysis mixture were added with ThT (20 µM) and samples were excited at 450 nm and 25 ± 0.1 °C. **b** Time-course fluorescence intensity of ThT at 482 nm during subtilisin-induced proteolytis of hTTR (black circles). The data points result from three different experiments. The time-course emission of ThT, in the presence of intact hTTR alone (without subtilisin), is reported as a control (white circles). The fluorescence signal is expressed as *F*/*F*_0_, where *F*_0_ is the emission intensity of ThT at time = 0 min. **c** Representative TEM micrograph of hTTR fibrils, generated after 72-h incubation of hTTR (0.2 mg/ml) with subtilisin at 37 °C in 20 mM potassium phosphate, pH 7.6, 100 mM KCl. **d** For comparison, a representative micrograph of hTTR fibrils, generated after incubation of intact hTTR (0.2 mg/ml) under acidic conditions (0.2 M potassium acetate buffer, pH 4.4) for 72 h at 37 °C is also shown. The scale bar (100 nm) is indicated.

The effect of hTTR cleavage by subtilisin on the formation of amyloid fibrils was monitored by turbidimetry and DLS (Fig. 4), ThT binding and transmission TEM (Fig. 5). At fixed time points, between 0 and 72 h, aliquots (50 µl) of hTTR-subtilisin proteolysis mixture (1 mg/ml) were withdrawn and immediately stored at −20 °C for biophysical analyses.

#### Turbidimetry

At increasing reaction time, the slope of the UV-absorption spectra of the hTTR proteolysis mixture remarkably increases, along with the value of *A*_260 nm_/*A*_280 nm_ ratio (Fig. 4a). These spectral features are consistent with the time-dependent formation of protein aggregates, scattering light with much higher intensity at lower wavelengths^39^. When the *A*_260 nm_/*A*_280 nm_ ratio (*r*) was plotted versus time, a sigmoidal curve was obtained, with a lag phase of about 10 h, when the turbidimetric signal remained essentially constant. After 24-h reaction of hTTR with subtilisin, the *r* value was enhanced by 30%, compared to that recorded at t = 0, and gradually increased up to 60% after 72-h reaction (Fig. 4b). For comparison, incubation of hTTR alone under identical experimental conditions (72-h incubation, without subtilisin), yielded a small increase of *r*, suggesting that hTTR undergoes some slow aggregation upon standing at pH 7.4. Blank measurements with subtilisin alone were also reported in Fig. 4a.

#### DLS

Considered that large molecules yield a DLS signal much more intense than small molecules, the % intensity size distribution plot was used as a convenient tool to detect even low amounts of hTTR aggregates in solution, before and after proteolysis with subtilisin. The %-intensity DLS plot of a freshly prepared hTTR solution shows a major peak (*R*_h_ = 3.8 ± 1.0 nm), corresponding to the tetrameric hTTR structure (peak A)^33^, and a minor peak (*R*_h_ = 82 ± 37 nm), corresponding to large hTTR aggregates (peak B) (Fig. 4c). However, when expressed as % mass size distribution, peak A accounts for >99% mass distribution, whereas peak B only for ~1% (Supplementary Fig. 2D and Table 1). After 24-h incubation with subtilisin, there is a remarkable size redistribution of hTTR species. The size of peak A is increased (*R*_h_ = 5.1 ± 2.0 nm), while peak B disappears and a new component is generated (*R*_h_ = 641 ± 465 nm), indicating the formation of much larger hTTR aggregates (peak C). Notably, after 24-h incubation of hTTR alone there is only a moderate shift of peak A and B to 4.3 ± 1.0 nm and 110.0 ± 62.0 nm, respectively, in agreement with turbidimetric data (Fig. 4a, b).

#### ThT binding assay

Whereas turbidimetric and DLS measurements monitor the formation of protein aggregates, ThT binding is a key indicator of the presence of ordered amyloid fibrils^39–41^ and is associated to a remarkable increase and a red shift of ThT absorption *λ*_max_ from 350 to 450 nm, along with an increased fluorescence quantum yield and a red shift of the maximum emission from 440 to 480 nm. Hence, the increase of ThT emission at 480 nm, after excitation at 450 nm, is taken as a “none-or-all” response for amyloid formation^40^.

The data in Fig. 5a indicate that, after 24-h reaction of hTTR (1 mg/ml) with subtilisin, there is a strong increase (>15 fold) of ThT emission intensity at 482 nm, indicative of ThT binding to the newly formed hTTR fibrils. Very similar results were obtained at 0.2 mg/ml hTTR concentration (Supplementary Fig. 6). Notably, 72-h incubation of ThT with hTTR alone yielded only <2-fold increase of ThT emission. As observed with turbidimetric measurements (Fig. 4b), the kinetics of fluorescence change exhibited a sigmoidal curve, with a similar lag phase of ~10 h, followed by a rapid growth and a final plateau (Fig. 5b). The sigmoidal shape of ThT binding kinetics is compatible with a nucleation-dependent aggregation model, where the lag phase corresponds to the nucleation step while the exponential part to fibril growth^42,43^.

#### TEM analysis

TEM analysis was carried out to confirm the generation of hTTR fibrils after 72-h incubation of hTTR (0.2 mg/ml) with subtilisin at 37 °C in TBS, pH 7.4. TEM micrographs (Fig. 5c) reveal the presence of irregular and wavy fibrils (50–100 nm long and 5–10 nm wide) characterized by a nodular morphology, typical of amyloid fibrils formed by intact hTTR^44–46^. For comparison, Fig. 5d shows TEM micrographs of hTTR fibrils obtained after incubating intact protein for 72 h at 37 °C under mild acidic conditions (pH 4.4), known to drive fibrillogenesis of hTTR^27^. As a negative control, any aggregate could not be detected after 72-h incubation of hTTR alone at 37 °C in TBS, at pH 7.4 (Supplementary Fig. 7). Our data indicate that addition of subtilisin to hTTR leads to the generation of hTTR(59–127) fragment, which starts forming aggregates that accumulate with time and display amyloid-like features.

### Amyloidogenic properties of purified hTTR(59–127)

**Fig. 6:**
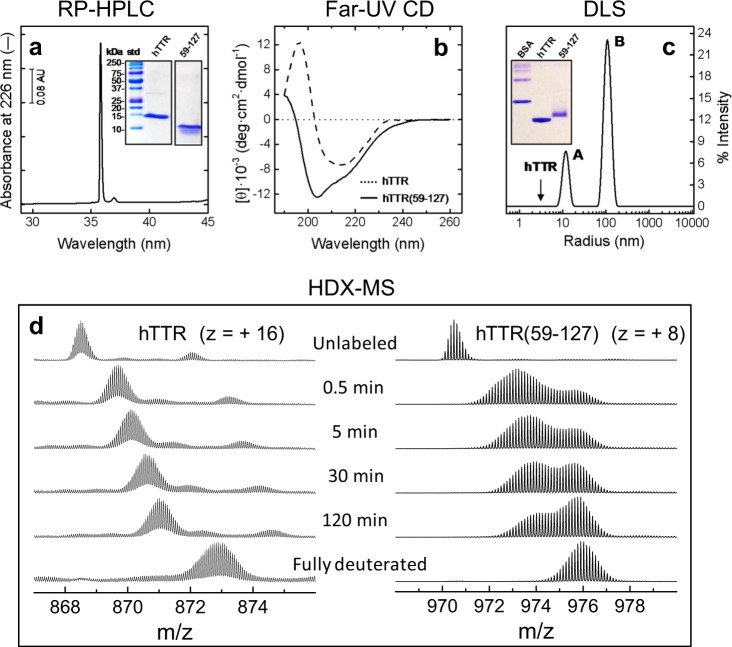
Purification and characterization of hTTR(59–127). **a** RP-HPLC purity check of hTTR(59–127). After lyophilisation, an aliquot of purified fragment was dissolved in 10 mM phosphate buffer, pH 7.4 and immediately injected onto an analytical C18 column, eluted (0.8 ml/min) with an acetonitrile-0.1%TFA gradient from 10 to 45% in 30 min. (Inset) SDS-PAGE (4–20% acrylamide) analysis of purified hTTR(59–127) (5 μg) under reducing conditions and Coomassie staining. For comparison, purified intact hTTR was also analysed. **b** Far-UV circular dichroism. Spectra of purified full-length hTTR and hTTR(59–127) fragment were recorded at a protein concentration of 0.1 mg/ml in 10 mM phosphate buffer, pH 7.4, at 25 °C. **c** DLS analysis. DLS measurements were carried out at a fragment concentration of 0.23 mg/ml. The arrow indicates the size of intact hTTR tetramer. DLS data are expressed as % intensity size distribution, while the values of *R*_h_, %mass distribution and %PD are reported in the Supplementary Table 1. All measurements were carried out at 25 °C, immediately after dissolving hTTR(59–127) protein lyophilisate with phosphate buffer. **Inset** Native PAGE (5–12% acrylamide) of hTTR and hTTR(59–127) fragment (5 μg). For comparison, BSA was also loaded. **d** HDX-MS kinetics of deuterium uptake by intact hTTR (left panel) and hTTR(59–127) fragment (right panel). Proteins were incubated at 25 °C with 90% D_2_O in PBS buffer, pH 7.4, and *m/z* spectra were taken at increasing labeling time. For intact hTTR, the multiple charged species (*z* = +16) of S-Cys-hTTR (MW = 13,880.8 Da) was considered, whereas for hTTR(59–127) the deuterium uptake of the *z* = +8 species was monitored. For hTTR at *t* = 0, the species with *m*/*z* = 872.05 corresponds to the S-Cys-Gly-hTTR isoform (i.e., species **2:i** in the Supplementary Fig. 1). A detailed description of the experimental procedure and data analysis is reported in the Supplementary Information. For clarity, the intensity of *m*_0_ and *m*_100_ spectra was downscaled by 4-fold, compared to the intensity of *m*_t_ spectra.

hTTR(59–127) was purified from the proteolysis mixture by semi-preparative RP-HPLC and thoroughly characterized in comparison with intact hTTR by denaturing and non-denaturing PAGE, far-UV circular dichroism, DLS, and global HDX-MS analysis (Fig. 6). The peptide material eluting with the major chromatographic peak (Fig. 6a) was collected, divided into aliquots, immediately frozen at −20 °C, and lyophilized. Fragment solutions were freshly prepared by dissolving lyophilized aliquots at 25 °C in 10 mM phosphate buffer, pH 7.4. Measurements were carried out soon after sample dissolution.

The far-UV circular dichroism spectrum of intact hTTR (Fig. 6b) is that typical of a protein containing high β-sheet content, consistent with the crystallographic structure of hTTR (i.e., 48% β-sheet content)^2^, whereas the spectrum of hTTR(59–127) (Fig. 6b) displays a prominent negative signal at 204 nm, indicative of a relative increase of the unfolded structure. DLS analysis of purified hTTR(59–127) (Fig. 6c) shows the presence of a two major species (peak A: *R*_h_ = 12.2 ± 2.0 nm; peak B: *R*_h_ = 110 ± 18 nm), larger than hTTR tetramer (*R*_h_ = 3.8 ± 1.0 nm) (Supplementary Fig. 2D and Table 1). Likewise, native PAGE (Fig. 6c, Inset) shows that hTTR(59–127) migrates slightly more slowly than intact hTTR. Altogether our results show that purified hTTR(59–127) forms aggregates which are looser or partially unfolded, compared to intact hTTR.

This conclusion is also corroborated by global HDX-measurements (Fig. 6d), showing that for intact hTTR the *m*/*z* centroid of the selected multiple-charged species gradually shifts to higher masses, with a burst-phase %D increase of ~25%. This trend is indicative of an EX2 exchange mechanism (Supplementary Fig. 8), which is typical of natively folded proteins undergoing fast local fluctuations at multiple sites^36,37^. At variance, for hTTR(59–127) the kinetics of deuterium uptake shows a bimodal distribution of H/D exchanging populations, whereby the unlabeled fragment is rapidly converted into two major species, i.e., *a* and *b*, where species *b* has a *m*/*z* value identical to that of the fully deuterated fragment. A burst-phase %D increase of ~51% was measured (Supplementary Fig. 8), indicative of a looser structure of the fragment. Species *a* then undergoes a gradual increase of *m*/*z* values and a concomitant (albeit non-quantitative) conversion to species *b*. These data are compatible with a mixed EX1–EX2 mechanism of exchange kinetics, and suggest that hTTR(59–127) aggregates are structurally heterogenous, containing either more compact and looser regions. More compact regions exchange with an EX2 mechanism, typical of folded proteins, while looser regions follow an EX1 mechanism, characteristic of partially structured polypeptides undergoing slow global unfolding reaction^36,37^.

**Fig. 7:**
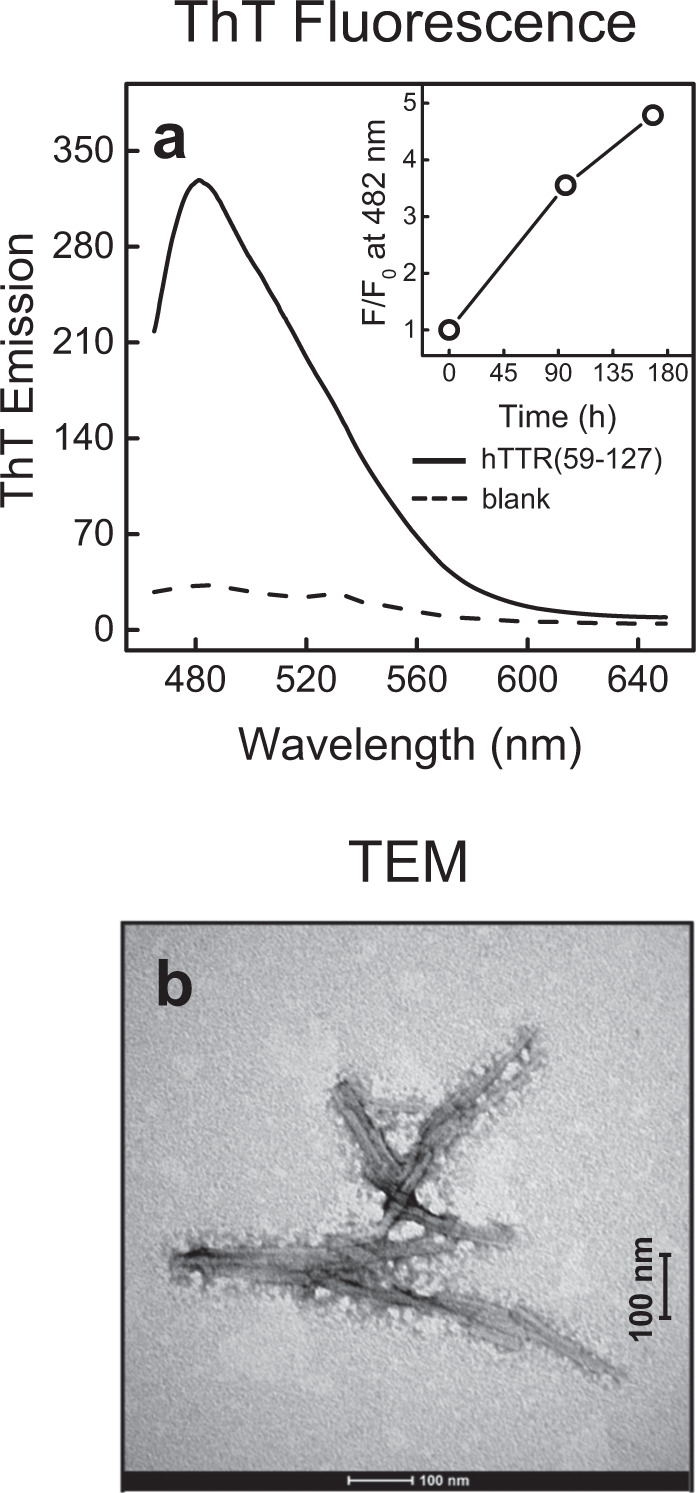
Analysis of hTTR(59–127) amyloid fibril formation. RP-HPLC purified fragment was lyophilized and dissolved (0.23 mg/ml) at 25 °C in TBS, pH 7.4. Amyloid fibril formation was tested by ThT binding assay and TEM soon after solubilization of the fragment in TBS. **a** ThT fluorescence emission in the presence (continuous line) or absence (dashed line) of hTTR(59–127). Measurements were carried out after 1-h incubation of ThT (20 µM) with hTTR(59-127) (50 μg/ml) and sample excitation at 450 nm. Inset: Time-dependent relative fluorescence emission (*F*/*F*_0_) of ThT (white circles) in the presence of hTTR(59–127), where *F* and *F*_0_ is the fluorescence intensity recorded after incubation with hTTR(59–127), as indicated. **b** Representative TEM micrograph of hTTR(59–127) solution. TEM analysis was carried out immediately after solubilization of lyophilized hTTR(59–127) at 0.6 mg/ml concentration. The average length and diameter of hTTR(59–127) fibrils was estimated as 200 ± 50 nm and 20 ± 5 nm, respectively. The scale bar (100 nm) is indicated.

When tested for ThT binding, purified hTTR(59–127) yielded a 10-fold increase of ThT fluorescence intensity at 482 nm, after excitation at 450 nm (Fig. 7a) and, after prolonged incubation (i.e., 160 h), ThT emission further increased by 5-fold (Fig. 7a, Inset). This result parallels ThT binding data obtained during proteolysis of hTTR with subtilisin (Fig. 5a) and demonstrates that the isolated fragment can spontaneously form amyloid-like aggregates. Finally, TEM analysis (Fig. 7b) provided conclusive evidence that isolated hTTR(59–127) forms ordered amyloid fibrils with typical rod-shaped morphology. Some amorphous protein material is also visible around the fibrils, likely corresponding to aggregates formed by looser/unstructured hTTR(59–127) monomers.

### Proteolysis of hTTR by subtilisin in human plasma

**Fig. 8:**
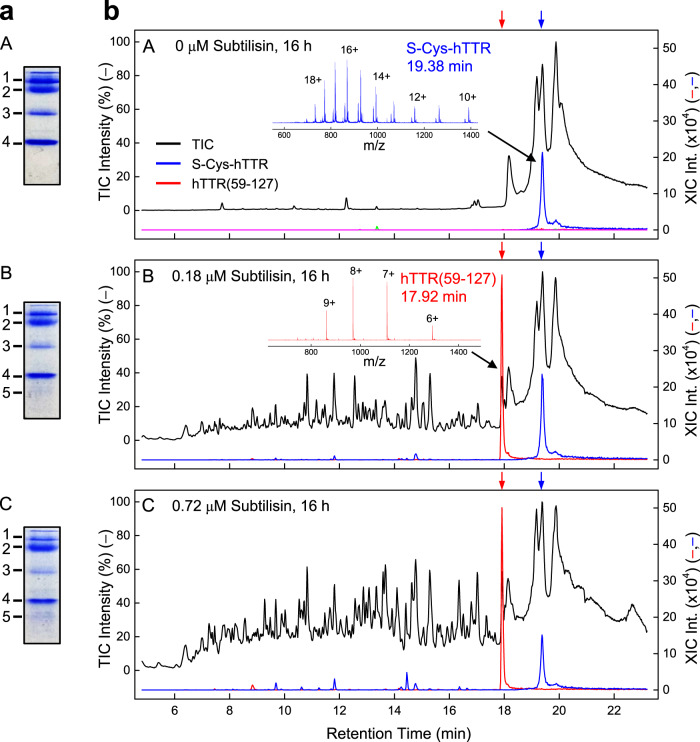
Generation of hTTR(59–127) in human plasma. Aliquots (800 μl) of human plasma were treated with subtilisin at different concentrations (0, 0.18, and 0.72 μM) at 37 °C under constant agitation (500 rpm). After 16-h reaction, plasma samples were processed following the phenol precipitation protocol (see “Methods” section) and analysed by SDS-PAGE and UPLC-MS. **a** SDS-PAGE (4–14% acrylamide) analysis under reducing conditions of phenol-treated plasma samples (8 μg). Proteins were identified by in situ tryptic digestion and peptide mass fingerprint analysis (see “Methods” section): 1, IgG heavy chain; 2, α_1_-acid-glycoprotein 1; 3, IgG light chain; 4, hTTR; 5, hTTR(59–127). **b** Representative UPLC-MS chromatographic profiles of phenol-treated plasma samples before (*A*) and after *(B, C)* 16-h incubation with increasing subtilisin concentrations. In each panel, the black trace reports the total ion current (TIC) profile, expressed in percentage, while the blue and red traces track the intensities of the highest *m*/*z* values of S-Cys-hTTR (*m/z* = 817.472) and hTTR(59–127) (*m/z* = 970.492) in the eXtracted Ions Chromatograms (XIC). The arrows refer to the retention times in the XICs of phenol-treated plasma samples spiked with hTTR (blue arrow) or hTTR(59–127) (red arrow).

Human plasma samples were incubated with subtilisin (0.18 and 0.72 μM) for 16 h at 37 °C. Most of plasma proteins were precipitated by addition of phenol^29^ while the residual intact hTTR and the newly generated hTTR(59–127) fragment were recovered in the supernatant, which was then analysed by SDS-PAGE and LC-MS (Fig. 8). In situ trypsin digestion and LC-MS analysis of the protein bands, resulting from proteolysis of human plasma with subtilisin, allowed us to identify intact hTTR and hTTR(59–127) fragment migrating as a weak/diffused band at lower molecular weights. Notably, the fragment band becomes more intense at higher substilisin concentrations, whereas it is absent in the plasma sample not treated with subtilisin. Likewise, after LC-MS analysis of subtilisin-treated plasma samples, we could safely identify residual intact hTTR and hTTR(59–127) by searching their multiple-charged species of highest intensity in the TIC trace (see the legend to Fig. 8). The extracted ion chromatograms (XICs) document that the peaks eluting at 17.9 ± 0.1 and 19.4 ± 0.1 min correspond to fragment hTTR(59–127) or intact S-Cys-hTTR isoform.

### Translocation of subtilisin across CaCo-2 cells monolayers

Subtilisin was reacted with fluorescein isothiocyanate (FITC) and only the mono-fluoresceinated derivative FTC-subtilisin was obtained. The proteolytic activity of FTC-subtilisin on Azocoll was found indistinguishable from the non-labeled protease^47^. FTC-subtilisin was also irreversibly inhibited at the active site with phenylmethylsulfonyl fluoride, to yield the inactive PMS-FTC-subtilisin. Labeled subtilisins were purified by size-exclusion chromatography and their chemical identity established by high-resolution MS (Supplementary Figure 9). As previously reported^47^, FTC-subtilisin could not be completely inhibited by PMSF, and our PMS-FTC-subtilisin preparation retained ~40% residual proteolytic activity (Supplementary Fig. 10). Monolayers of CaCo-2 cells were prepared in Transwell polyester membrane cell culture inserts (Fig. 8a). Monolayer integrity and development of functional tight junction complexes were verified by Transepithelial Electrical Resistance measurements^48^.

**Fig. 9:**
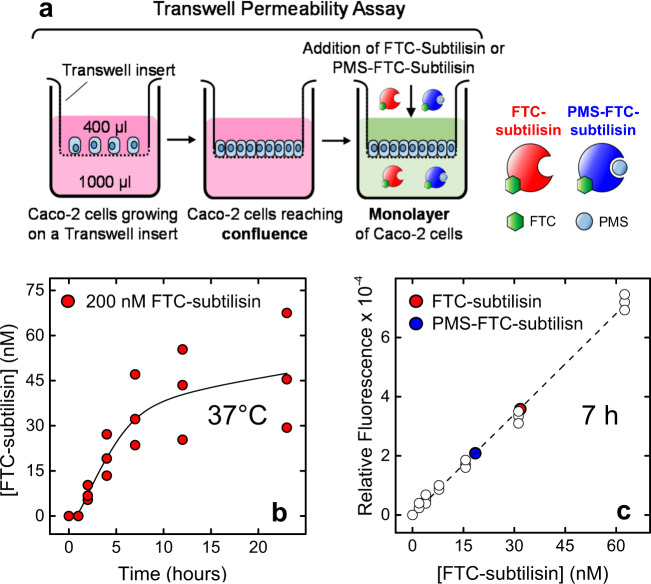
Caco-2 cells permeability assay of fluoresceinated subtilisin. **a** Schematic representation of Caco-2 cells permeability assay. Monolayers of Caco-2 cells on the Transwell permeable inserts were prepared as detailed in the “Methods” section. Subtilisin translocation was assessed at 37 °C by adding catalytically active FTC-subtilisin or inactive PMS-FTC-subtilisin (0.2 µM, 400 µl), in 10 mM 2-[4-(2-hydroxyethyl)piperazin-1-yl]ethanesulfonic acid, pH 7.4, 10 mM glucose, on the apical chamber and measuring the intensity of fluorescein emission in the basolateral chamber at increasing translocation times. Blank experiments were carried out in the absence of subtilisin. **b** Translocation kinetics of FTC-subtilisin (red circles) across Caco-2 monolayers. **c** The concentration of translocated labeled subtilisin was determined using a calibration curve obtained with known concentrations of labeled enzyme (withe circles). Red and blue circles refer to the concentration of FTC-subtilisin and PMS-FTC-subtilisin, respectively, found in the basolateral chamber of the Transwell apparatus after 7-h translocation. Samples were excited at 488 nm and the fluorescence intensity was measured at 535 nm, after baseline subtraction. The data points are the results of three different experiments.

Active FTC-subtilisin (200 nM) was added to the apical chamber and the rate of translocation at each time point estimated by measuring the intensity of fluorescein emission in the basolateral chamber (Fig. 9b), using a suitable calibration curve (Fig. 9c). The chemical integrity of labeled subtilisin, after translocation across CaCo-2 cells monolayers, was verified by high-resolution MS (Supplementary Fig. 11). The kinetics of FTC-subtilisin translocation follows a sigmoidal trend, characterized by: (i) a lag-phase (0–1 h), in which no (or negligible) transepithelial passage occurs; (ii) a burst-phase (1–7 h), where translocation markedly increases with time; (iii) a nearly plateau-phase (7–24 h), where there is a gradual (albeit small) increase of translocation rate. Notably, after 7-h incubation, the translocation rate of active FTC-subtilisin (16%) was almost 2-fold higher than that of the inactive PMS-FTC-subtilisin (9%) (Fig. 9c).

## Discussion

Fragmented hTTR is the standard composition of fibrils in both ATTR^9,19–23^ and SSA^19,24,25^, where a mixture of C-terminal fragments is variably represented, with fragments starting at positions from 44 to 59^19,20,22–25^. While the chemical heterogeneity of fragmented hTTR in amyloid deposits emphasizes the role of proteolytic enzymes in hTTR amyloidosis, the protease(s) responsible for abnormal cleavage of hTTR in vivo has(ve) yet to be safely identified^9,26^. Noteworthy, the generation of amyloidogenic fragments from soluble precursor proteins is not restricted to hTTR amyloidosis but also involves Alzheimer’s^49,50^ and Parkinson’s^51^ disease.

Bellotti and co-workers have recently reported that fragment 49–127, often found in amyloidogenic deposits in vivo, can be generated in vitro by incubating recombinant wild-type hTTR or hTTR variants (e.g., Ser52Pro and Val30Met) with the digestive protease trypsin^9,52^ or with the fibrinolytic enzyme plasmin^26^. Notably, proteolysis reaction was conducted at high shear stress, i.e., double-orbital shaking to 900 rpm, which is thought to mimic the mechanical forces acting on plasma proteins in flowing blood^52^. In the absence of shaking, proteolysis of amyloidogenic mutants by trypsin or plasmin is much less efficient^9,26,52^ and wild-type hTTR becomes fully resistant to these proteases^9,26,52^. Hence, both destabilizing mutations and shear stress induce mild/partial unfolding of hTTR tetramer, driving the formation of misfolded monomers which are more easily cleaved to generate amyloidogenic fragments. Regarding the possible involvement of trypsin in SSA, Bellotti and co-workers outlined that trypsin is secreted from the exocrine pancreas exclusively into duodenal lumen, and therefore it is thought not to encounter hTTR in human blood^9,52^. Furthermore, the concentration of active plasmin in SSA patients (i.e., typically elderly subjects >75 years) is decreased by age-related overexpression of proteinase inhibitors, such as plasminogen activator inhibitor-1 and α_2_-antiplasmin^53,54^. Notably, cleavage by trypsin was further confirmed using the synthetic hTTR peptide 41-60 and its Lys48Ala analog, encompassing the cleavage site Lys48-Thr49^55^.

Starting from these considerations, we decided to screen several different proteolytic enzymes for their hTTR-cleaving ability. To this aim, natural hTTR was challenged under static/stagnant conditions in physiological buffer with digestive, coagulative, fibrinolytic, neutrophil, and bacterial proteases. These enzymes belong to different protease families and display very different substrate specificities and catalytic mechanisms. Among the 15 proteases tested, only subtilisin from *B. subtilis* proved to efficiently cleave hTTR in vitro (Fig. 1) and generate fragment hTTR(59–127), which was resistant to further proteolysis and accumulated over time to form amyloid-like fibrils (Fig. 5). Notably, neither trypsin nor plasmin cleaved hTTR (Fig. 1), consistent with recent findings reported by Bellotti and colleagues with trypsin on recombinant wild type hTTR^9,52^.

Next, we checked whether subtilisin could cleave endogenous hTTR in human plasma, a complex *milieu* containing high concentrations (0.5–2.9 mg/ml) of protein protease inhibitors (e.g., α_1_-proteinase inhibitor and α_2_-macroglubulin), acting as scavengers of potentially harmful proteases, e.g., trypsin, elastase, or thrombin, being generated during key physiological processes, such as coagulation, innate immunity, and inflammation^56^. These inhibitors lack stringent specificity and therefore can potentially inhibit subtilisin and impair hTTR cleavage in vivo. To rule out this possibility, subtilisin (180 nM) was incubated for 16 h at 37 °C with freshly prepared human plasma and the generation of hTTR(59–127) investigated by LC-MS analysis (Fig. 8). Noteworthy, our data clearly indicate that substantial amounts of the amyloidogenic fragment hTTR(59–127) were almost exclusively generated. These results provide clear-cut evidence that, even after prolonged incubation in human plasma, subtilisin can escape inactivation by protease inhibitors in vivo and cleave plasma hTTR. Importantly, the same fragment hTTR(59–127), deriving from both wild-type and Val30Met mutant hTTR, was indentified in vivo in the amyloid deposits of patients with ATTR^22,23^.

*B. subtilis* is a Gram-positive bacterium lacking obvious pathogenicity and abundantly present as a commensal in the gut of normal individuals (i.e., 10,000 spores/g of feces)^57^. *B. subtilis* secretes eight extracellular proteases, but the majority of its proteolytic activity can be ascribed to subtilisin and neutral protease (NP), which account for about 75% and 25% of the total *B. subtilis* extracellular proteolytic activity, respectively^58^. Subtilisin, the prototype of the subtilisin-like protease family members, is an alkaline serine protease characterized by very broad substrate specificity, ranging from aromatic to basic and even acidic amino acids, with preference for large uncharged residues at the primary specificity site^34^. Like subtilisin, NP lacks substrate specificity, with some preference for apolar amino acids. NP is a zinc-dependent metallo-protease, belonging to the thermolysin-like proteinase subfamily and, conversely with subtilisin, it is not able to cleave hTTR (Fig. 1).

At this point the question arises as to whether subtilisin can unfold its hTTR-cleaving activity also in vivo and act as a pro-amyloidogenic protease in hTTR amyloidosis. To answer this question, and keeping in mind that subtilisin cleaves hTTR and generates hTTR(59–127) in human plasma, it is crucial to establish whether subtilisin can translocate across the intestinal mucosa and reach the bloodstream to accomplish its hTTR-cleaving activity. Hence, subtilisin was first labeled with fluorescein and then the permeability of the gut mucosa to active FTC-subtilisin assessed on a CaCo-2 cell simulated intestinal epithelium, along with the (partially) inhibited PMS-FTC-subtilisin. Although CaCo-2 cells derive from human epithelial colorectal adenocarcinoma and the cell model lacks some physiological factors that might influence the absorption of macromolecules (e.g., the unstirred water layer and the presence of mucus and food), CaCo-2 cell monolayers recapitulate the morphological and functional properties of normal intestinal (absorptive) enterocytes. Indeed, they are widely exploited by pharmaceutical companies and regulatory authorities as a standard permeability-screening assay for predicting intestinal permeability and in vivo absorption in humans^48^. The data in Fig. 9d, e indicate that substantial amounts of active FTC-subtilisin can translocate across the CaCo-2 cell simulated intestinal epithelium (i.e., up to 45% after 24-h incubation). The translocation rates measured for FTC-subtilisin are identical to those reported by others on a similar Caco-2 cells system for digestive (trypsin and chymotrypsin) and plant (bromelain and papain) proteases of comparable size^59,60^. The sigmoidal curve of FTC-subtilisin translocation kinetics is compatible with a self-enhanced paracellular diffusion mechanism^59,60^, whereby active proteases first degrade intercellular tight junctions (i.e., the lag-phase) and then diffuse across the epithelium (i.e., the burst-phase) until transmembrane equilibrium concentration is reached (i.e., the plateau-phase). PMS-FTC-subtilisin displays about 40% of the hydrolytic activity of uninhibited FTC-subtilisin and, noteworthy, a proportionally lower translocation rate was measured (Fig. 9e), indicating that proteolytic loosening of tight junctions between CaCo-2 cells in the epithelial monolayer is required for protease translocation^59^.

Although our findings seem to challenge the traditional notion that intestinal mucosa is an impenetrable barrier to the uptake of intraluminal large macromolecules, they are in keeping with numerous biochemical and clinical evidences, accumulated during the last 40 years, showing that considerable (albeit small) amounts of proteins and enzymes can be absorbed in their intact and biologically active form^59,60^.

Although ATTR is one of the most frequent hereditary protein misfolding diseases worldwide, only about 10,000 cases of genetic ATTR have been estimated in the global population^7,8,16^. At variance, acquired SSA has been recently recognized as a common, albeit underappreciated, cause of cardiomyopathy and heart failure in older adults, with an estimated prevalence as high as 1–3% of elderly people >75 years of age (see also “Introduction” section)^12–18^. Whereas the pathogenesis of genetic ATTR can be rationalized considering the effects that amino acid mutations have on hTTR tetramer structure and its susceptibility to proteolysis^26–28^, the cause of the late onset of SSA in elderly people is much more difficult to decipher and the molecular events leading to the late onset of cardiac hTTR amyloid deposits in older patients remains mysterious, despite normal hTTR circulating in their blood from birth^26^. All these considerations strongly suggest that non-genetic, age-related factors must play a role in the pathogenesis of SSA.

Importantly, ageing is positively (and variably) related with increased permeability of the intestinal mucosa (often referred to as “leaky gut”), moderate chronic inflammatory state, and intestinal microbiota dysbiosis^61,62^. These changes are reciprocally connected, whereby alteration of the intestinal microbial composition increases intraluminal protease activity and, as a result, boosts self-enhanced paracellular permeability of the intestinal mucosa through proteolytic degradation of intercellular tight junction proteins or activation of protease-activated receptor 2 receptors^63–66^. The selective passage of (macro)molecules from the gut lumen to the systemic circulation is regulated by the Gut-Vascular Barrier, an anatomical/functional structure mainly formed by epithelial intestinal and endothelial vascular cells^67^. Notably, under normal conditions, the cut-off threshold molecular weight of Gut-Vascular Barrier is around 40–70 kDa^67^, higher than that of subtilisin (27 kDa), and it can be variably enhanced by age-related factors (e.g., gut microbiota dysbiosis, low-grade inflammation) or in patients with Inflammatory Bowel Diseases and intestinal infections^63,67^.

To the best of our knowledge, identification of subtilisin as a hTTR-cleaving protease, potentially able to translocate across the intestinal barrier, puts forward a novel pathogenetic mechanism for SSA. Indeed, the increased permeability of the gut mucosa in some predisposed individuals (e.g., older adults) may allow subtilisin, and perhaps other (yet unidentified) bacterial hTTR-cleaving proteases, to pass across the intestinal mucosa into the bloodstream and generate hTTR amyloidogenic fragments, which can deposit as infiltrative fibrils in the heart of SSA patients. The model well accounts for the late and age-dependent onset of SSA in elderly population^12–18^ and is consistent with recent work showing that patients with hTTR amyloidosis present higher plasma proteolytic activity^68^. The pathogenetic mechanism highlighted in this work also allows to reconsider under a physio-pathological perspective the data of hTTR proteolysis by trypsin, obtained in vitro at high shear stress^9,52^. Active trypsin of pancreatic origin, indeed, has been found in the ileum^69^ from which it is partially reabsorbed^70^. Therefore, pathological increase of intestinal permeability may result into abnormally high blood trypsin concentrations and generation of the amyloidogenic fragment 49–127, ubiquitous in hTTR amyloid deposits^19,20,22–25^.

The pathological significance of subtilisin-hTTR cleavage system and the relevance of gut permeability to proteases, highlighted in this work, pave the way to devise suitable therapeutic strategies aimed at controlling harmful increase of proteolytic activity in the intestine and restore the integrity of the Gut-Vascular Barrier.

## Methods

### Reagents

Coagulative and fibrinolytic proteases were purchased from Hematologic Technologies (Essex Junction, VT, USA), while all other proteases were from Athens Research-Technology (Athens, GA, USA) or Sigma (Saint Louis, MO, USA). *B. subtilis* neutral protease was a generous gift from Dr. Guido Grandi (EniRicerche, Milan, Italy). All other salts, solvents and reagents were of analytical grade and purchased from Sigma or Merck (Darmstadt, Germany).

### Purification and chemical characterization of hTTR from human plasma

Natural hTTR was purified following the phenol precipitation method previously reported^29^, with minor modifications. Plasma samples from blood donors and non-smokers healthy subjects were obtained from the Institutional Blood Bank of the University Hospital of Padua and the Institute of Experimental Medicine, Saint Petersburg, Russia. All subjects gave their informed consent to the present study. Chemical modifications at Cys10 in purified hTTR were identified by LC-MS, with a Waters (Milford, MO, USA) Xevo G2-S Q-TOF mass spectrometer and a Waters Acquity H-Class UPLC system. Separations were carried out on a C4 or C18 microbore column (Grace-Vydac, Columbia, MD, USA) eluted with a water:acetonitrile-1%(v/v) formic acid gradient. Mass analyses were run in the positive ion mode, with the capillary potential set at 1.5 kV and source temperature at 100 °C. Monoisotopic mass values were determined at a resolution >35,000 and an accuracy <5 ppm. Data were acquired with the Mass-Lynx 4.1 software and analysed with the BioPharmaLynx 1.3.4 suite (Waters). The oligomeric state of purified hTTR was determined by analytical size-exclusion chromatography on a Yarra SEC-3000 column (Phenomenex, Torrance, CA, USA). The apparent molecular weight of hTTR was determined using a calibration curve obtained with standard proteins, as previously described^71^.

### Proteolysis of hTTR

hTTR samples (100 μl; 1 mg/ml) in TBS, pH 7.4, containing 5 mM CaCl_2_ (TBS-CaCl_2_) were incubated at 37 °C in the presence of the selected protease, at a protease:hTTR molar ratio of 1:20. At time intervals, aliquots (10–20 µg) of each proteolysis mixture was acid quenched and analysed by reducing SDS-PAGE (4–15% acrylamide and Coomassie staining) and RP-HPLC. The chemical identity of the proteolytic fragments was established by LC-MS analysis, as above. Quantitative determination of intact hTTR and hTTR(59–127) fragment was performed by densitometric analysis of the electrophoretic gel bands, or by integrating the area under the chromatographic peaks. Kinetic data of hTTR degradation were analysed according to the pseudo-first order reaction model^72^. For preparative purposes, hTTR(59–127) was purified by semi-preparative RP-HPLC. After lyophilisation, the purified fragment was dissolved in 10 mM phosphate buffer, pH 7.4, and tested for amyloid formation.

Proteolysis of hTTR in human plasma was conducted by incubating plasma samples (800 μl) with increasing concentrations of subtilisin at 37 °C for 16 h, under static or dynamic conditions (500 rpm) using a Thermomixer Compact (Eppendorf-AG, Hamburg, Germany). Human plasma was obtained from the Institutional Blood Bank of the University Hospital of Padua. Residual intact hTTR and the newly generated hTTR(59–127) were recovered by the phenol precipitation method (see above). Intact hTTR and hTTR(59–127) were identified by reducing SDS-PAGE (4–14% acrylamide, Coomassie staining) and UPLC-MS after in situ tryptic digestion of the corresponding gel bands and peptide mass fingerprint analysis^71^. S-Cys-hTTR isoform and hTTR(59–127) fragment were identified by searching the multiple-charged species of highest intensity in the total ion current (TIC) trace. The extracted ion chromatograms (XICs) were obtained from the TIC plots using the DisplayMass tool, as implemented in the MassLynx software.

### HDX-MS measurements

Mass spectra were acquired in the resolution mode (*m*/*z* 50–2000) on a Waters Xevo G2S, equipped with a standard electrospray ionization source. For global exchange analysis, the kinetics of deuterium incorporation into intact proteins was determined by measuring %*D* at increasing incubation times with 90% D_2_O at 25 °C, as previously detailed and according to the following equation^36,37^: *%D* = *(m*_t_ *– m*_0_)/(*m*_100_ − *m*_0_), where *m*_t_ is the mass of the protein after labeling time *t*, *m*_0_, and *m*_100_ are the mass of the protein before and after extensive deuteration, respectively, taking into account back-exchange effects that were minimized by thermostating at 0 °C the eluents, the injection valve, and the column. MaxEnt software was used to deconvolute *m/z* spectra.

For local exchange analysis^36,37^, samples were prepared by incubating hTTR (3 μM) with 90% D_2_O in PBS, pH 7.1, at 22 °C. At time points, aliquots (30 μl) were withdrawn and acid quenched at 0 °C in 0.2 M sodium phosphate buffer, pH 2.4, containing 1.5 M guanidinium hydrochloride and on-line digested at 20 °C with pepsin using a Poroszyme Immobilized Pepsin Cartridge (Applied Biosystems, Foster City, CA, USA). The peptides eluting from the Pepsin Cartridge were on-line trapped, concentrated, desalted on a C18 VanGuard BEH Precolumn and then fractionated with an Acquity UPLC BEH C18 column (Waters) eluted with a water-acetonitrile-0.1% formic acid. Each peptide was identified in the MS^E^ mode, using argon as collision gas. Data were processed using the BioPharmaLynx suite (Waters) and each fragmentation spectrum was manually inspected to confirm the reliability of the automated assignment. The HDExaminer vs. 2.5 software (Sierra Analytics, Modesto, CA, USA) was used to calculate %*D* values for the selected fragments and then to generate the heatmap of %*D*. All HDX measurements were conducted in triplicate with the error as the standard deviation.

### Spectroscopic measurements

Protein concentration was determined spectrophotometrically on a Jasco (Tokyo, Japan) instrument at 280 nm, using an absorptivity (*ε*_0.1%_) value of 1.35 mg^−1^ cm^2^ for intact hTTR and 1.67 mg^−1^ cm^2^ for hTTR(59–127). The time-course aggregation of hTTR(59–127) was monitored by recording the turbidimetric signal^39^ of the proteolysis mixture of hTTR (18 μM as tetramer) with subtilisin (0.9 μM) at 37 °C in TBS-CaCl_2_, as the absorbance ratio (Abs_260_/Abs_280_) in the UV spectrum. Circular dichroism spectra were taken on a Jasco J-1500 spectropolarimeter, equipped with a Peltier temperature control system^73^. Circular dichroism signal was expressed as the mean residue ellipticity [*θ*] = *θ*_obs_·MRW/(10·l·c), where *θ*_obs_ is the observed signal in degrees, MRW is the protein mean residue weight, *l* is the cuvette pathlength in cm, and c is the protein concentration in g/ml. Fluorescence spectra were recorded on a Jasco FP-6500 spectrofluorimeter, equipped with a Peltier temperature control system. Amyloid fibril formation was assessed by time-dependent ThT fluorescence binding assay^39^. DLS measurements were performed in TBS on a Zetasizer Nano-S instrument (Malvern Instruments, UK). DLS measurements yield the values of *R*_h_ and %PD, where *R*_h_ is the hydrodynamic radius, i.e., the radius of a hard sphere that diffuses at the same rate as that of the molecule considered, and %PD is the polydispersity index, i.e., the width of the particle size distribution of a protein in a sample^74^. Scattering data were analyzed with the Nano-6.20 software.

### TEM measurements

TEM analyses were carried out by placing a drop of the sample solution in 20 mM potassium phosphate, pH 7.6, 0.1 M KCl, on a Butvar-coated copper grid (TAAB-Laboratories Equipment Ltd, Berks, UK), air dried and negatively stained with 1.0% (w/v) uranyl acetate solution. TEM micrographs were taken on a Tecnai G2 12 Twin instrument (FEI Company, Hillsboro, OR, USA) using an excitation voltage of 100 kV.

### Caco-2 cells permeability assay

Fluoresceinated subtilisin was prepared as previously detailed^47^ and characterized by MS and Azocoll protease assay. Caco-2 cells (American Type Culture Collection, Manassas, VA, USA) were grown in high-glucose Dulbecco’s modified Eagle’s media. Cells were trypsinized and seeded in the upper chamber of Transwell polyester membrane cell culture inserts. Medium was replaced in upper and lower chambers every 24 h. After 14 days, the formation of confluent cellular monolayers with well-developed tight junctions was confirmed by Transepithelial Electrical Resistance measurements. Labeled subtilisins in the same medium were added to the apical chamber of the Transwell inserts. Aliquots were collected from the basolateral compartment at 2-h intervals, placed in a 96-wells plate, and excited at 488 nm, while the emission intensity was measured at 535 nm. Subtilisin translocation was quantified using a calibration curve obtained with known concentrations of labeled enzyme. Blank permeability experiments were performed with Dulbecco’s modified Eagle’s media.

### Statistics and reproducibility

Proteolysis experiments, chromatographic separations, mass spectrometry and spectroscopic measurements, and CaCo-2 cells permeability assays were independently replicated three times and individual data points are reported for each experiment.

## Supplementary information

Supplementary Information

Description of Additional Supplementary Files

Supplementary Data 1

## Data Availability

All source data underlying the graphs and charts presented in the main figures are available in the Supplementary Data 1. Uncropped images of the coomassie blue-stained electrophoresis gels and TEM micrographs are presented in the Supplementary Figs. 12 and 13. All other data that support the findings of this study are available from the corresponding author upon reasonable request.
