# Supplementary material for: A serine protease secreted from Bacillus subtilis cleaves human plasma transthyretin to generate an amyloidogenic fragment
Source: Commun Biol. 2020 Dec 11;3:764. doi: 10.1038/s42003-020-01493-0 (PMC7733459; doi:10.1038/s42003-020-01493-0)
Supplement: Supplementary file 1 — Supplementary Information [file 42003_2020_1493_MOESM1_ESM.pdf]

# Supplementary Information

## A serine protease secreted from *Bacillus subtilis* cleaves human plasma transthyretin to generate an amyloidogenic fragment

Daniele Peterle [1,a], Giulia Pontarollo [1,b], Stefano Spada [1,b], Paola Brun [2], Luana Palazzi [1], Alexej V. Sokolov [3,4], Barbara Spolaore [1,5], Patrizia Polverino de Laureto [1,5], Vadim B. Vasilyev [3,4], Ignazio Castagliuolo [2], and Vincenzo De Filippis [1,5] *

[1] Department of Pharmaceutical and Pharmacological Sciences, University of Padua, Padua, Italy; [2] Department of Molecular Medicine, University of Padua, Padua, Italy; [3] Institute for Experimental Medicine, Saint Petersburg, Russia; [4] Saint Petersburg University, Saint Petersburg, Russia; [5] CRIBI Biotechnology Center, University of Padua, Padua, Italy.

[a] *Current address*: Dept. of Chemistry and Chemical Biology, Northeastern University, 360 Huntington Ave. 02115, Boston, MA, USA

[b] *Current address*: Center for Thrombosis and Hemostasis (CTH) University Medical Center Mainz, Langenbeckstraße 1, 55131 Mainz, Germany

Supplementary Table 1. DLS analysis of hTTR and hTTR(59-127) [a]

| | hTTR (0 h) [b] | | | hTTR (24 h) [c] | | | hTTR + Subtilisin (24 h) [d] | | | hTTR(59-127) [e] | | |
|---|---|---|---|---|---|---|---|---|---|---|---|---|
| #Peak | $R_h$ (nm) [f] | % Int [g] | % PD [h] | $R_h$ (nm) | % Int | % PD | $R_h$ (nm) | % Int | % PD | $R_h$ (nm) | % Int | % PD |
| A | 3.8 ± 1.0 | 53.3 | 24.6 | 4.3 ± 1.0 | 63.3 | 28.4 | 5.1 ± 2.0 | 75 | 35.7 | 12.2±2 | 22.5 | 13.7 |
| B | 82.1 ± 37.2 | 46.7 | 41.5 | 110.1 ± 62.0 | 36.7 | 48.2 | - | - | - | 110±18 | 77.5 | 41.5 |
| C | - | - | - | - | - | - | 640.7 ± 465.3 | 25 | 59.4 | - | - | - |

[a] Data are relative to DLS measurements in Fig. 4C and Fig. 6C and are expressed as % intensity size distribution. [b, c] DLS data of intact hTTR before and after 24-h incubation in TBS buffer, pH 7.4, containing 5 mM $CaCl_2$. [d] DLS data of the proteolysis mixture of hTTR with subtilisin after 24-h reaction. [e] DLS data of purified hTTR(59-127). [f] $R_h$ is the hydrodynamic radius, with standard deviation (±). [g] % intensity of the considered species. [h] % polydispersity.

Supplementary Table 2. UPLC-MS analysis of the proteolysis reaction of hTTR with subtilisin [a].

| Peak ID # [b] | Fragment mass [c] (a.m.u.) | Fragment Sequence [e] |
|---|---|---|
| 1 | 337.17 (337.17) [d] | R104-Y105 |
| 2 | 268.11 (268.11) | S77-Y78 |
| 3 | 454.22 (454.22) | G53-H56 |
| 4 | 365.16 (365.16) | S112-Y114 |
| 5 | 557.28 (557.28) | G22-N27 |
| 6 | 332.18 (332.18) | W79-K80 |
| 7 | 856.46 (856.46) | A120-E127 |
| 8 | 582.28 (582.28) | S77-K80 |
| 9 | 351.18 (351.18) | G67-Y69 |
| 10 | 1347.69 (1347.66) | G4-V16 |
| 11 | 478.24 (478.24) | L111-Y114 |
| 12 | 500.27 (500.27) | V30-F33 |
| 13 | 982.41 (982.41) | T59-E66 |
| 14 | 315.22 (315.21) | A109-L111 |
| 15 | 516.31 (516.31) | W79-L82 |
| 16 | 519.27 (519.27) | G83-F87 |
| 17 | 766.41 (766.40) | S77-L82 |
| 18 | 577.25 (577.25) | W41-F44 |
| 19 | 979.39 (979.39) | A37-F44 |
| 20 | 855.40 (855.42) | E63-Y69 |
| 21 | 7162.74 (7162.74) | F64-E127 |
| 22 | 7291.78 (7291.78) | E63-E127 |
| 23 | 7420.78 (7420.82) | E62-E127 |
| 24 | 7549.80 (7549.85) | E61-E127 |
| **25** | 7751.90 (7751.87) | T59-E127 |

[a] Proteolysis and UPLC-MS analysis were carried out as detailed in the Methods. [b] Peak ID refers to the elution order of the proteolytic fragments from the UPLC column, as estimated from the TIC trace. [c] Experimental monoisotopic mass values are reported for each fragment. [d] Theoretical monoisotopic mass values of the proteolytic hTTR fragments, as obtained from the PeptideMass tool https://web.expasy.org/peptide_mass/. [e] Assigned sequence of hTTR proteolytic fragments. The entire hTTR sequence was covered, except the N-terminal fragment 1-3 of the protein, most likely eluting with the void volume from the RP-column.

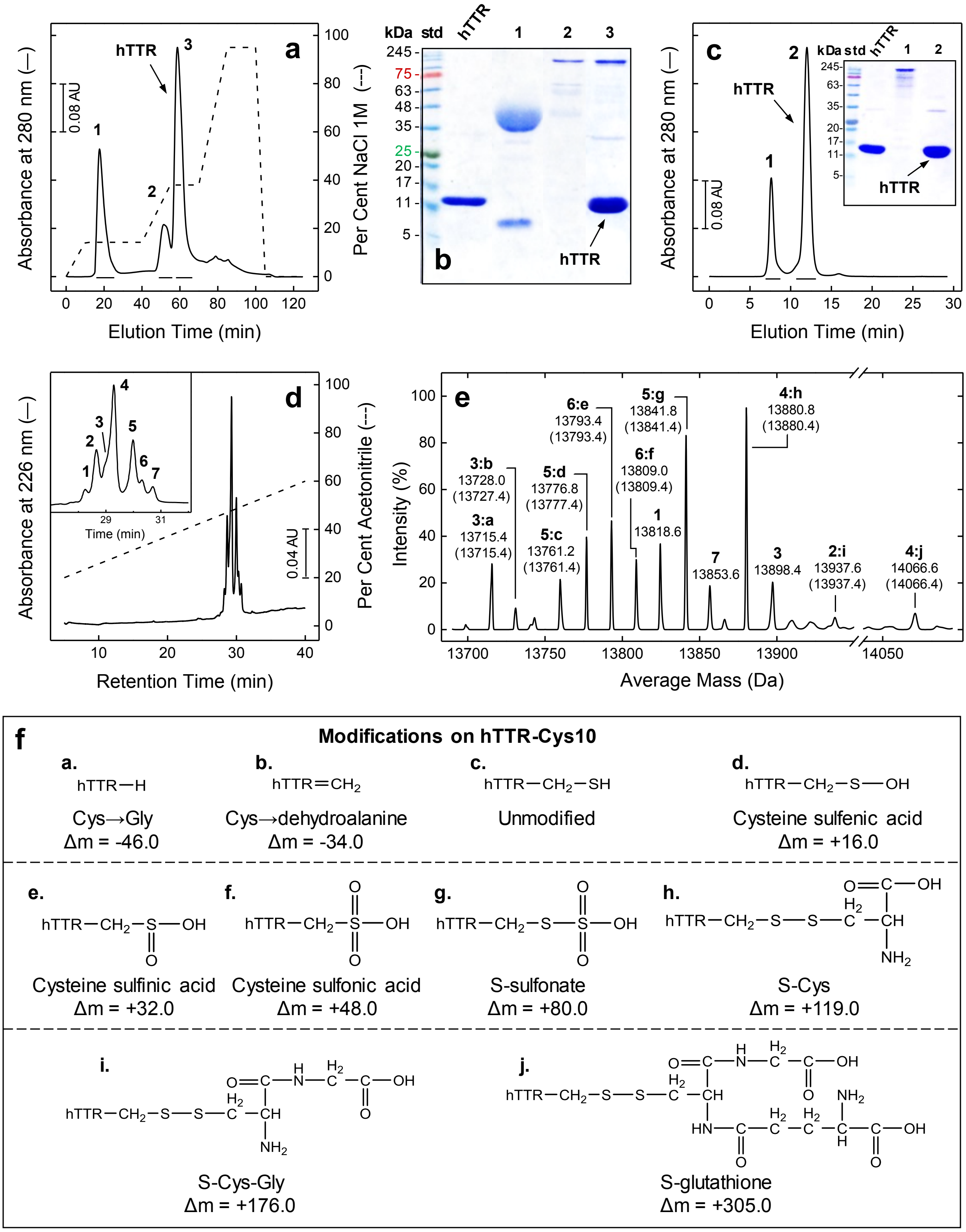

a
hTTR
Absorbance at 280 nm (—)
Per Cent NaCl 1M (---)
0.08 AU
Elution Time (min)
b
kDa
std
hTTR
1
2
3
245
75
63
48
35
25
20
17
11
5
c
kDa std hTTR 1 2
Elution Time (min)
d
Absorbance at 226 nm (—)
Per Cent Acetonitrile (---)
0.04 AU
Time (min)
Retention Time (min)
e
Intensity (%)
Average Mass (Da)
3:a 13715.4 (13715.4)
3:b 13728.0 (13727.4)
5:c 13761.2 (13761.4)
5:d 13776.8 (13777.4)
6:e 13793.4 (13793.4)
6:f 13809.0 (13809.4)
1 13818.6
5:g 13841.8 (13841.4)
7 13853.6
4:h 13880.8 (13880.4)
3 13898.4
2:i 13937.6 (13937.4)
4:j 14066.6 (14066.4)
f
Modifications on hTTR-Cys10
a. Cys→Gly Δm = -46.0
b. Cys→dehydroalanine Δm = -34.0
c. Unmodified
d. Cysteine sulfenic acid Δm = +16.0
e. Cysteine sulfinic acid Δm = +32.0
f. Cysteine sulfonic acid Δm = +48.0
g. S-sulfonate Δm = +80.0
h. S-Cys Δm = +119.0
i. S-Cys-Gly Δm = +176.0
j. S-glutathione Δm = +305.0

**Supplementary Fig. 1 Purification and chemical characterization of human plasma hTTR. a** Anion exchange chromatography. After phenol precipitation and centrifugation, the supernatant solution containing hTTR was loaded onto a Q-Sepharose column, equilibrated with 10 mM Tris-HCl, pH 7.4 and eluted with a gradient of NaCl (---) at a flow rate of 1.0 ml/min. **b** Reducing SDS-PAGE (4-12% acrylamide) analysis of the fractions 1, 2, and 3 (10-µl aliquots) eluted from the Q-Sepharose column in panel A; std: molecular weight protein standard; hTTR: commercial hTTR from human plasma. **c** Size exclusion chromatography. An aliquot of fraction 3 in panel A was loaded onto a Superose-12 column, eluted with 5 mM Tris·HCl buffer, pH 7.4, 0.15 M NaCl, containing 5 mM $CaCl_2$. **Inset** Reducing SDS-PAGE (4-12% acrylamide) analysis of the fractions (10-µl aliquots) eluted from the Superose-12 column in panel C. **d** RP-HPLC analysis of purified hTTR. An aliquot (20 µg) of fraction 2 in panel C was loaded onto an analytical C4 column, eluted with a linear acetonitrile-0.078%TFA gradient (---) at a flow rate of 0.8 ml/min. **Inset** Close-up view of the RP-HPLC chromatogram. The numbers near the chromatographic peaks indicate the fractions that were collected and further analysed by mass spectrometry. **e** Representative deconvoluted mass spectrum of hTTR purified by SEC, as in panel C. An aliquot (1 µg) of fraction 2 was added with 1:1 water:acetonitrile solution (10 µl) containing 1% (v/v) formic acid and loaded onto a Xevo G2S Q-TOF mass spectrometer. The numbers and letters near the mass peaks indicate the elution order of each species in the RP-HPLC chromatogram in panel **d** (Inset) and the corresponding chemical modification reported in panel F, respectively. For instance, the notation **5:g** identifies the species eluting as peak **5** in panel **d** and containing the modification **g** at Cys10, i.e. the S-sulfonate derivative. Mass values are reported as average mass and the numbers in parenthesis refer to the theoretical values of each hTTR species. **f** Chemical modifications of hTTR at Cys10, as reported[1]. $\Delta$m values refer to the mass differences existing between each modified hTTR species and the unmodified hTTR.

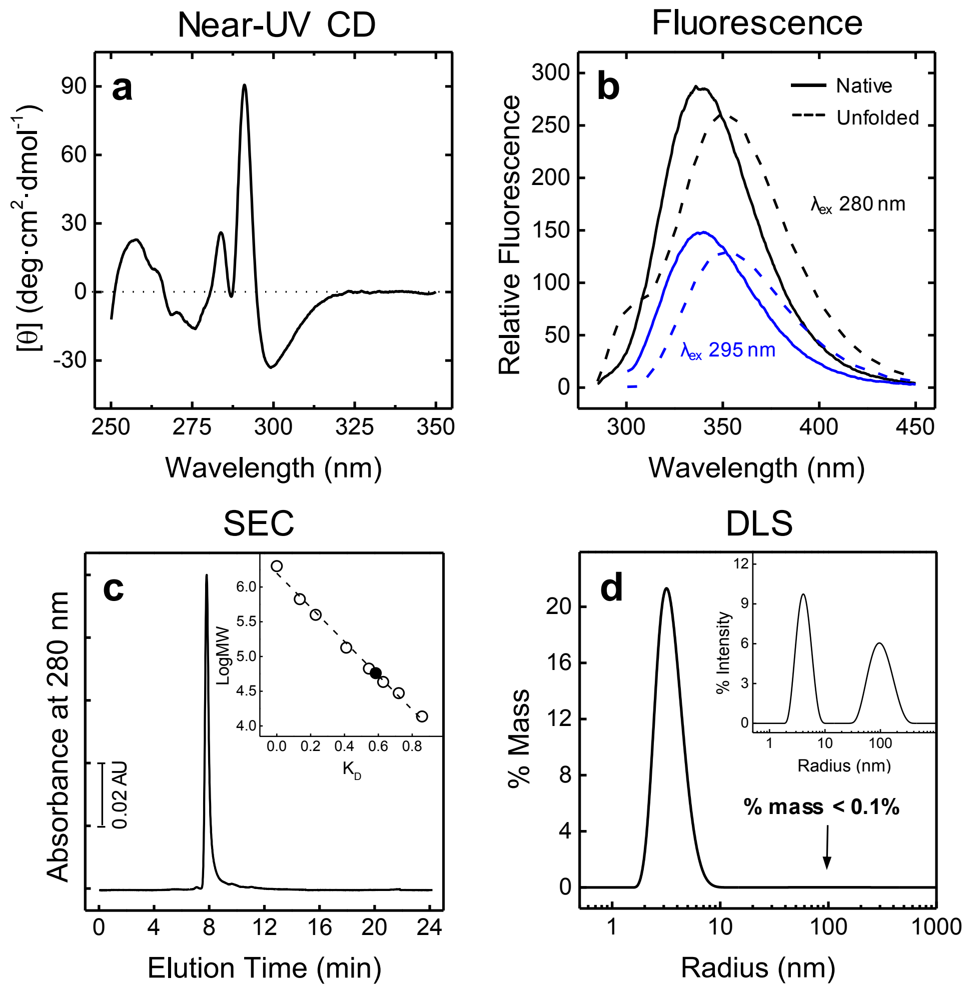


**Supplementary Fig. 2 Conformational characterization and oligomerization state of purified hTTR.** **a** Near-UV circular dichroism spectrum of purified hTTR (1.5 mg/ml), under native conditions. **b** Fluorescence spectra of purified hTTR (25 μg/ml), under native and denaturing conditions, after excitation at 280 nm (black lines) and 295 nm (blue lines). All measurements were carried out at 25.0 ± 0.1°C, after baseline subtraction, under native (**—**, 10 mM sodium phosphate buffer, pH 7.4) and denaturing (---, 10 mM sodium phosphate buffer, pH 7.4, 7 M Gnd-HCl) conditions. **c** SEC analysis of hTTR. An aliquot (10 µl) of a purified hTTR stock solution (1 mg/ml) was loaded onto a Yarra SEC-3000 column, eluted with 20 mM Tris-HCl buffer, pH 6.8, 0.15 M NaCl at a flow-rate of 0.6 ml/min. **Inset** Calibration curve of the Yarra SEC-3000 column (LogMW = 2.51 ± 0.07 - 0.40 ± 0.01·$K_D$, R = 0.99) obtained with standard proteins (○): blue dextran (2.000 kDa), thyroglobulin (669 kDa), apoferritin (443 kDa), BSA dimer (134 kDa) and monomer (67 kDa), ovoalbumin (43 kDa), carbonic anhydrase (29 kDa), and RNase-A (13.7 kDa). A molecular weight of 57 ± 3 kDa was estimated for hTTR (●), consistent with the protein tetrameric structure. **d** DLS analysis of hTTR. Prior to analysis, hTTR samples (1 mg/ml, 50 µl) were equilibrated for 30 min at 25°C in 5 mM Tris·HCl buffer, pH 7.4, 0.15 mM NaCl. DLS data are expressed as % mass size distribution, with a $R_h$ = 3.8 ± 1.0 nm and % PD = 24.6. **Inset** DLS trace of hTTR expressed as % intensity size distribution. An additional component is present, with a $R_h$ = 82.1 ± 37.2 nm and % PD = 41.5%.

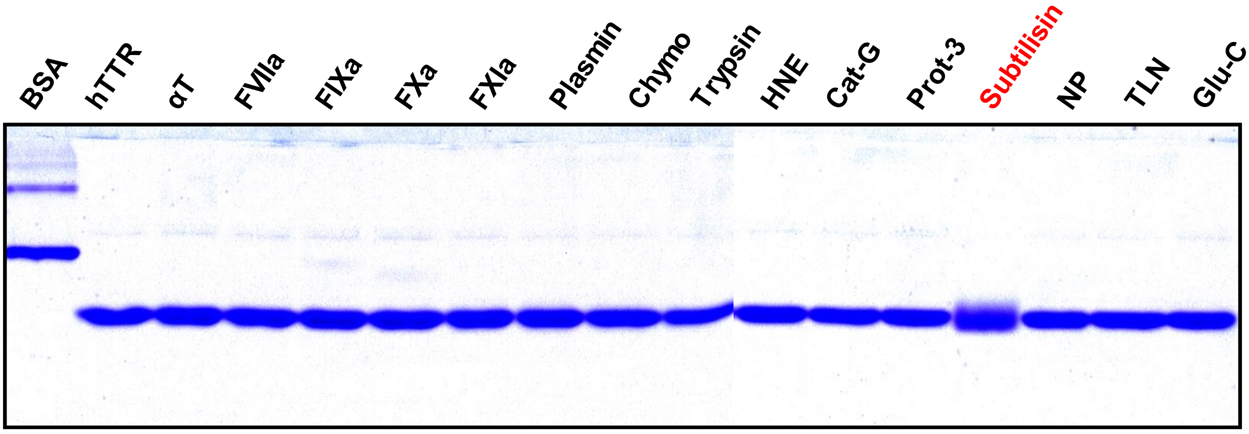


**Supplementary Fig. 3 Native PAGE analysis of hTTR proteolysis by different proteases.** hTTR (1 mg/ml) was reacted at 37°C in TBS pH 7.4, containing 5 mM $CaCl_2$, with different proteases at an enzyme:hTTR ratio of 1:20 (mol/mol). After 24-h reaction, aliquots (7 μg) the proteolysis mixtures were analysed by native PAGE (5-12% acrylamide) and Coomassie stained. For comparison, an aliquot of BSA was also loaded.

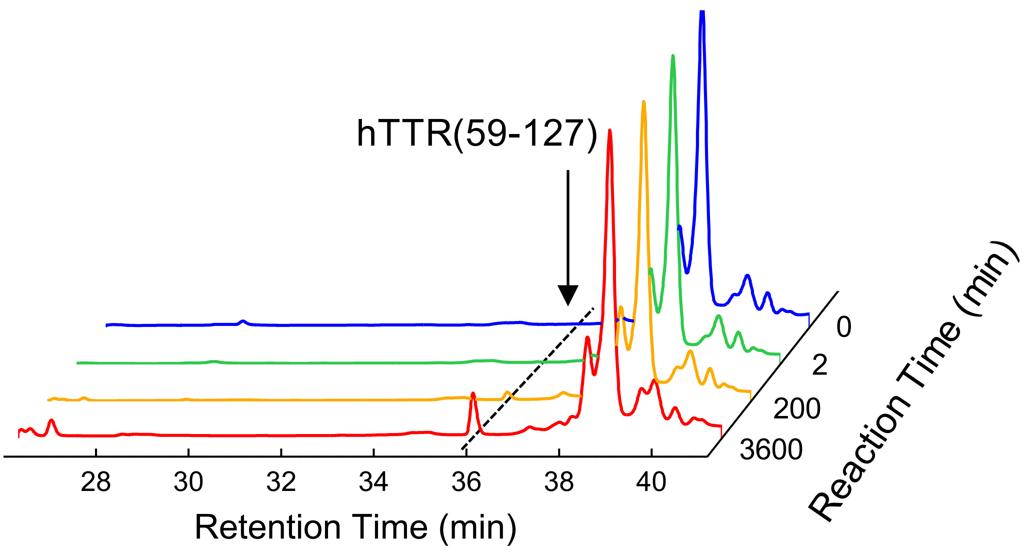


**Supplementary Fig. 4 Time-course RP-HPLC analysis of hTTR proteolysis by subtilisin.** hTTR (1 mg/ml) was reacted in TBS, pH 7.4, containing 5 mM $CaCl_2$, with subtilisin (0.09 μM) at an enzyme/hTTR molar ratio of 1:200 at 37°C. At increasing time points, the proteolysis mixtures were acid-quenched and aliquots (20 μg) analysed by RP-HPLC. RP-HPLC waterfall plot of hTTR proteolysis by subtilisin. Aliquots of the reaction mixture were loaded onto a C18 analytical column and eluted with a linear aqueous acetonitrile-0.1% TFA gradient from 10% to 45% in 30 minutes, at a flow rate of 0.8 ml/min. The absorbance of the effluent was recorded at 226 nm. The material eluted in correspondence of the chromatographic peaks were collected and identified by MS analysis.

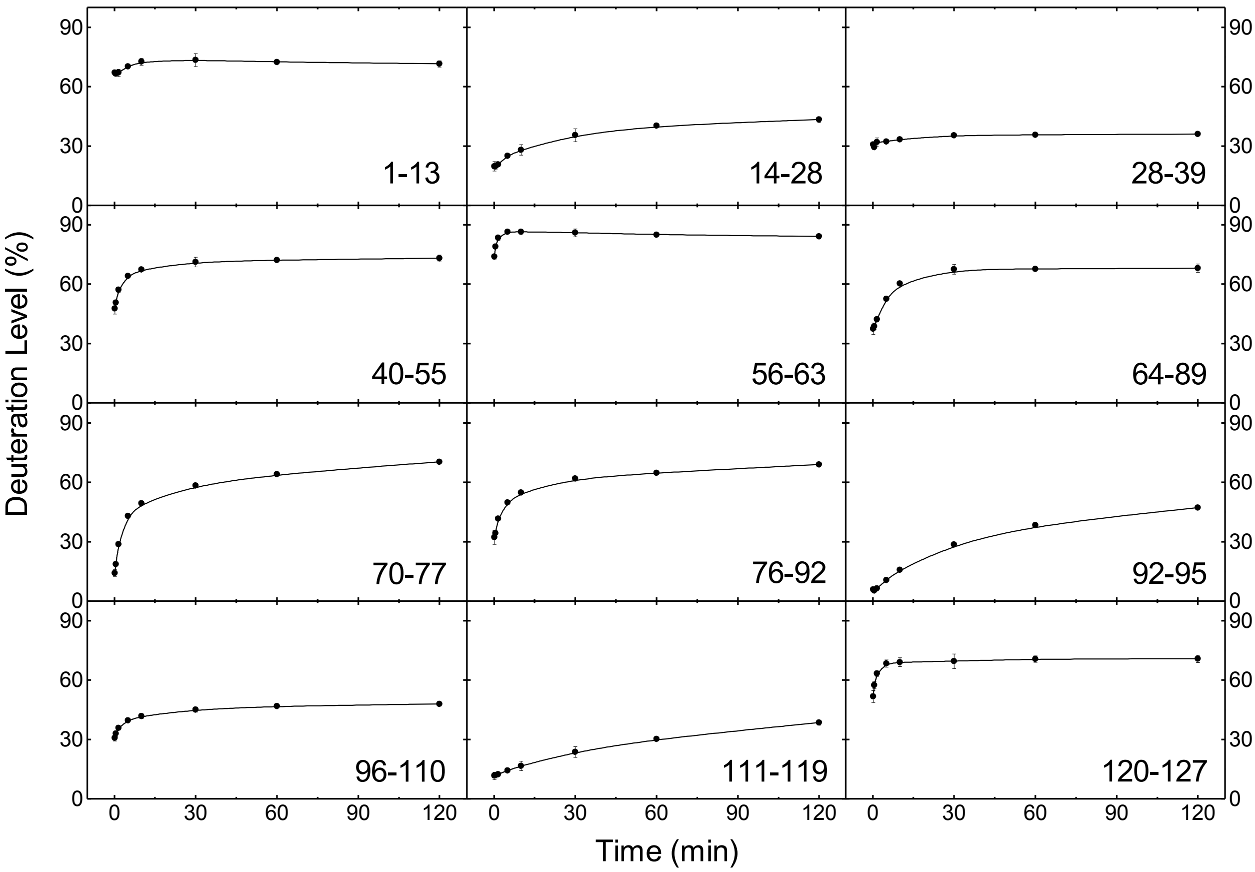


**Supplementary Fig. 5 Time-course analysis of deuterium uptake by hTTR peptic fragments**. The peptides derive from hTTR digestion with pepsin and were selected to cover the entire hTTR sequence. HDX-MS measurements were conducted in triplicate and the error bars represent the standard deviation. Fitting curves are solely intended to follow the HDX profile.

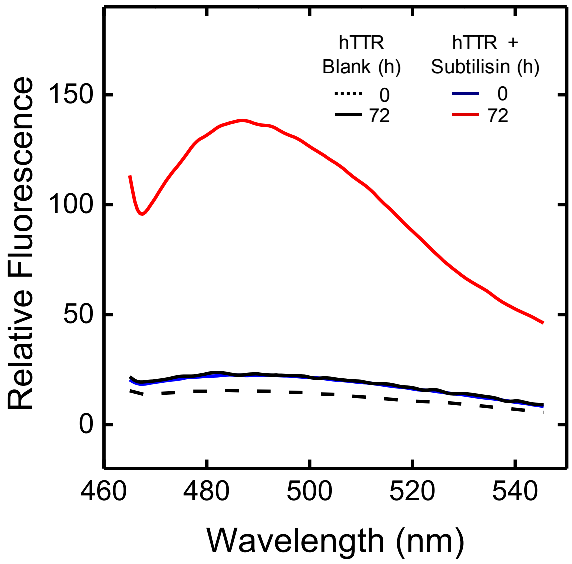


**Supplementary Fig. 6 ThT binding assay of amyloid fibril formation during proteolysis of hTTR with subtilisin.** hTTR (0.2 mg/ml) was treated at 37 °C with subtilisin in TBS buffer, pH 7.4, 5 mM $CaCl_2$ at an enzyme:hTTR ratio of 1:20 (mol/mol). At time points, aliquots (50 µl) of the proteolysis reaction were withdrawn, diluted ten-fold with the same buffer, and analysed by ThT binding assay. At the indicated time points, aliquots of the proteolysis mixture were added with ThT (20 µM) and samples were excited at 450 nm and 25 ± 0.1°C.

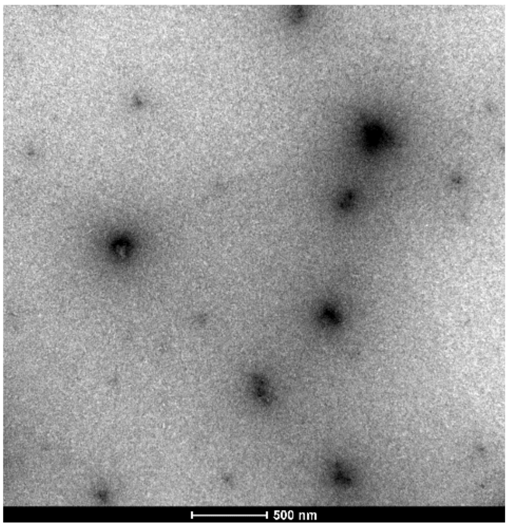


**Supplementary Fig. 7 Representative TEM micrograph of isolated hTTR**. Images were obtained after incubating hTTR alone (0.2. mg/ml) for 72 h at 37°C in TBS, at pH 7.4. The scale bar (500 nm) is indicated.

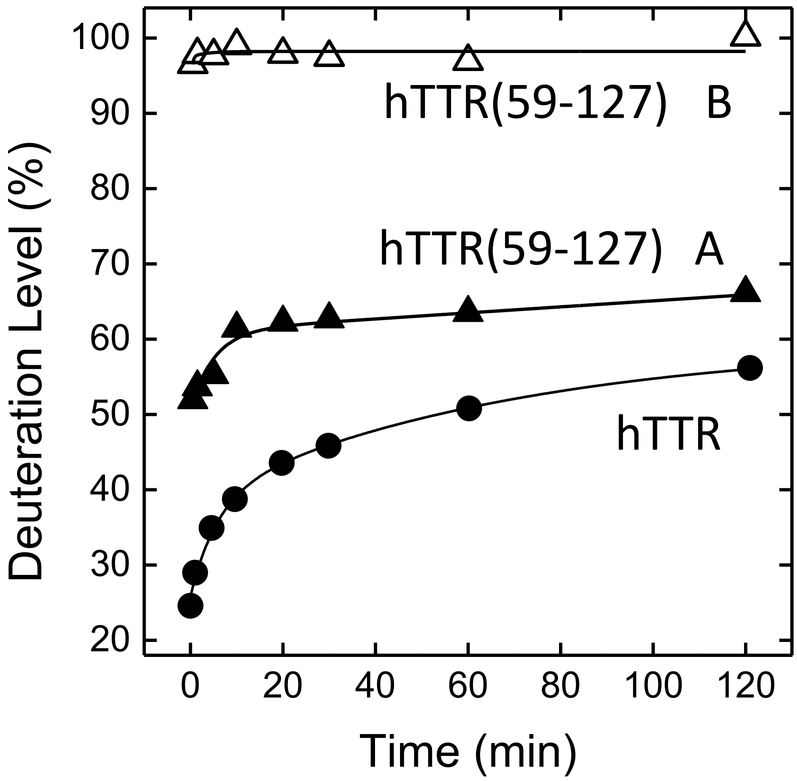


**Supplementary Fig. 8 HDX-MS kinetic analysis of global deuterium uptake by plasma purified hTTR**. Measurements were carried out in TBS, pH 7.4. The data points are the average of three different experiments, with error bars as the standard deviations. The unlabelled and fully deuterated protein yield m/z values of 869.72 (13899.6 ± 0.8 a.m.u) and 872.84 (13949.4 ± 0.9 a.m.u), respectively. The data were interpolated with the equation describing a biexponential HDX labelling[2,3]: $\%D = A_0 + A_1[1 - exp(-k_1t)] + A_2[(1 - exp(-k_2t)]$, where $A_0$ is the fraction of hydrogen ions that undergo burst phase exchange with deuterium ions in the dead time of the experiment; $A_1$ and $A_2$ are the fractions of hydrogen ions that have been exchanged with the apparent rate constants $k_1$ and $k_2$, respectively. Non-linear regression of HDX kinetic data was carried out using Origin vs. 9.0 software (MicroCal Inc. MA, USA). Best-fit parameters were obtained as follows: $A_0$ = 25.1 ± 1.0 %, $A_1$ = 21.0 ± 3.6 %, $k_1$ = 0.015 ± 0.009 $min^{-1}$, $A_2$ = 13.7 ± 2.6 %, $k_2$ = 0.18 ± 0.05 $min^{-1}$, $A_{max}$ = 59.7 ± 1.5 %.

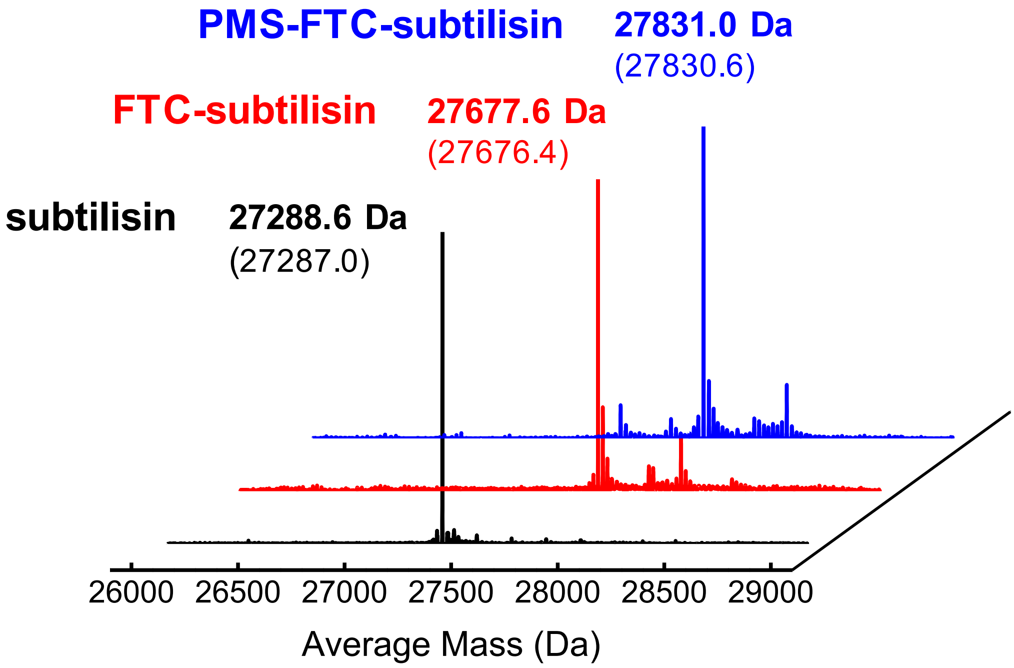


**Supplementary Fig. 9 High-resolution MS spectra of purified subtilisin, FTC-subtilisin and PMS-FTC-subtilisin**. The numbers in parenthesis refer to the theoretical average mass values of subtilisin derivatives. Analyses were carried out as detailed in the Methods.

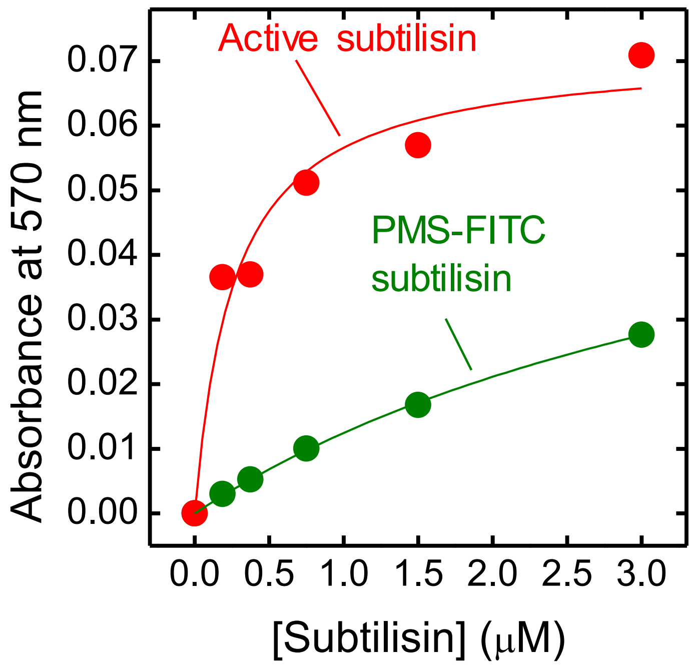


**Supplementary Fig. 10 Residual activity of PMS-FTC-subtilisin.** Aliquots (100 μl) of active FTC-subtilisin or PMS-FTC-subtilisin (from 50 μM to 24 nM, 1:2 v/v serial dilutions) solution in PBS (10 mM $Na_2HPO_4$ pH 7.4, 0.15 M NaCl) were added to 400 μl of freshly prepared Azocoll suspension (1.5 mg/ml in PBS) and incubated with a gentle end-to-end rotation at room temperature. After 2-h incubation, the suspension was centrifuged (10,000 x g, 10 min, 25°C) and the supernatant (200 μl) samples were transferred to a 96-well microtiter plate. Residual activity was quantified by measuring the fluorescence intensity at 570 nm.

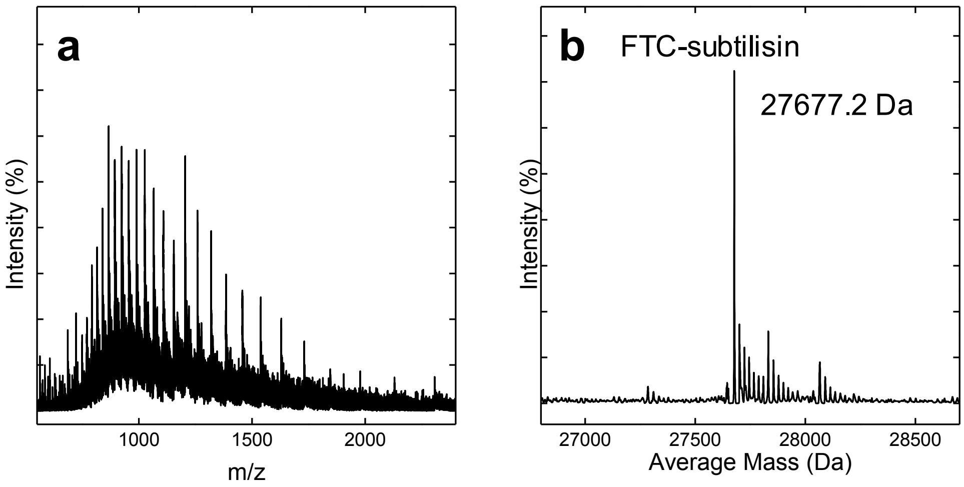


**Supplementary Fig. 11 Mass spectrometry analysis of FTC-subtilisin in the Caco-2 cells permeability assay.** After 7-h translocation in the Transwell apparatus, an aliquot of solution in the basolateral chamber was collected and analysed by LC-MS on a Xevo G2S spectrometer, yielding the theoretical molecular mass (27676.4 Da) of intact FTC-subtilisin. **a** mass/charge (m/z) spectrum and **b** deconvoluted mass spectrum of translocated FTC-subtilisin.

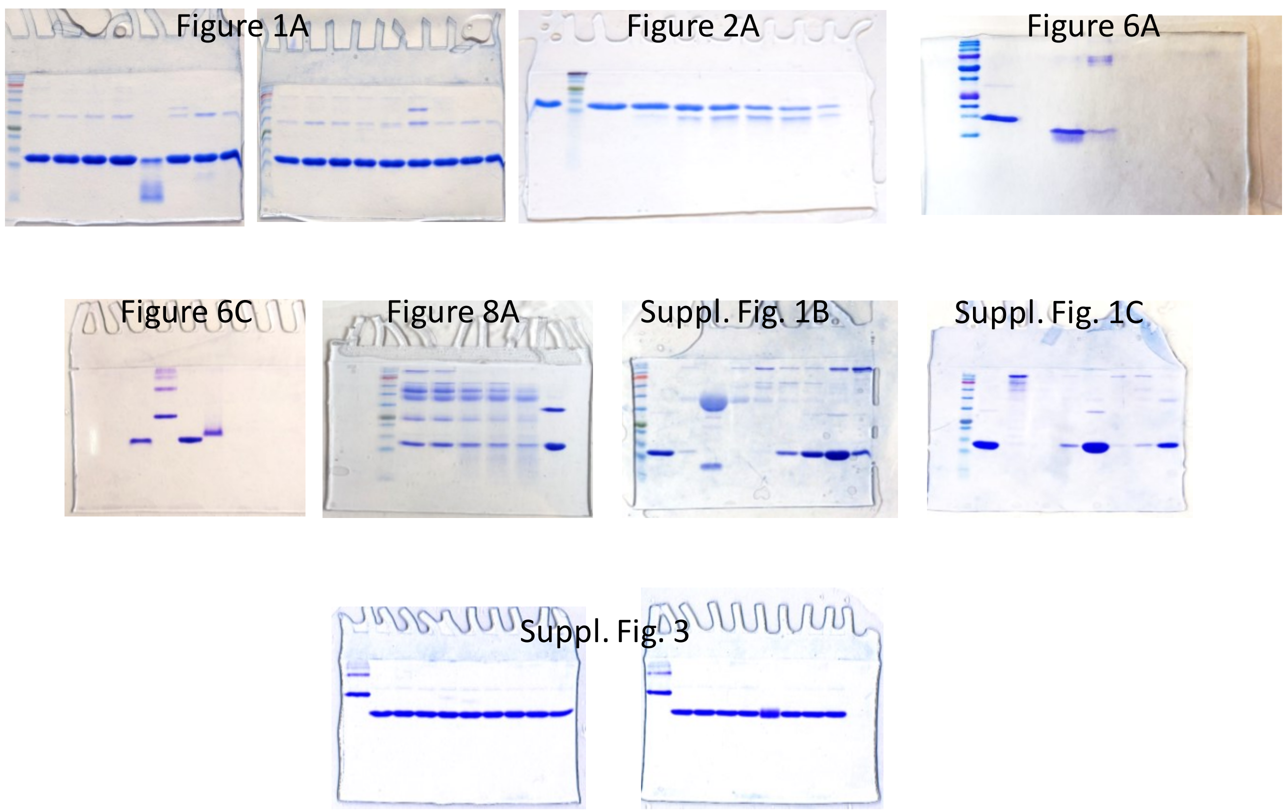


**Supplementary Fig. 12 Uncropped images of Coomassie-stained gels**. Labels indicate the corresponding figures where these images are found in the main text or in the supplementary materials.

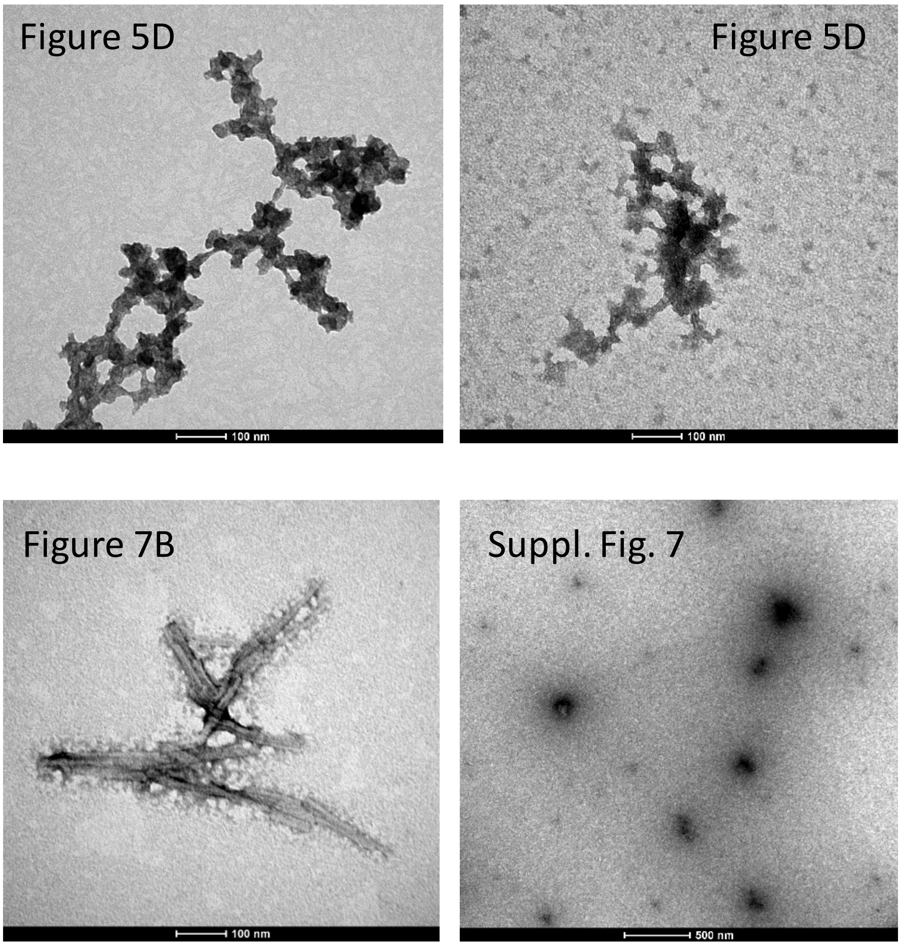


**Supplementary Fig. 13 Uncropped images of TEM micrographs**. Labels indicate the corresponding figures where these images are found in the main text or in the supplementary materials.

## Supplementary Methods

**Reagents.** Subtilisin Carlsberg (EC-3.4.21.62) from *B. subtilis*, thermolysin (EC-3.4.24.27) from *B. thermoproteolyticus*, endoproteinase Glu-C (EC-3.4.21.19), trypsin (EC-3.4.21.4), chymotrypsin (EC-3.4.21.1), and horse heart apomyoglobin were purchased from Sigma (Saint Louis, MO, USA). Plasmin (EC-3.4.21.7), α-thrombin (EC-3.4.21.5), coagulation factors VIIa (EC-3.4.21.21), IXa (EC-3.4.21.22), Xa (EC-3.4.21.6), and XIa (EC-3.4.21.27) were purchased from Haematologic Technologies (Essex Junction, VT, USA). Proteinase 3 (EC-3.4.21.76), cathepsin G (EC-3.4.21.20) and human neutrophil elastase (EC-3.4.21.37) were from Athens Research-Technology (Athens, GA, USA), while *B. subtilis* neutral protease (EC-3.4.24.28) was a generous gift from Dr. Guido Grandi (EniRicerche, Milan, Italy). Horse heart apo-myoglobin was prepared from holo-myoglobin by heme extraction with 2-butanone. FITC, PMSF and Azocoll were from Sigma. $D_2O$ (99.90% v/v) was from EurIsotop (Saint-Aubin, France). All other salts, solvents and reagents were of analytical grade and purchased from Sigma or Merck.

**Purification and chemical characterization of hTTR from human plasma.** Natural hTTR was purified following the phenol precipitation method previously reported[4], with minor modifications. Plasma samples from blood donors and non-smokers healthy subjects were obtained from the Institutional Blood Bank of the University Hospital of Padua and the Institute of Experimental Medicine, Saint Petersburg, Russia. All subjects gave their informed consent to the present study. Chemical modifications at Cys10 in purified hTTR were identified by LC-MS, with a Waters (Milford, MO, USA) Xevo G2-S Q-TOF mass spectrometer and a Waters Acquity H-Class UPLC system. Separations were carried out on a (1 x 50 mm) C4 or C18 microbore column (Grace-Vydac) eluted (50 μl/min) with a water:acetonitrile-1%(v/v) formic acid gradient. Mass analyses were run in the positive ion mode, with the capillary potential set at 1.5 kV and source temperature at 100°C. Monoisotopic mass values were determined at a resolution >35.000 and an accuracy <5 ppm. Data were acquired with the Mass-Lynx 4.1 software and analysed with the BioPharmaLynx 1.3.4 suite (Waters).

The oligomeric state of purified hTTR was determined by analytical SEC on a Yarra SEC-3000 column (Phenomenex, CA, USA). The apparent molecular weight of hTTR was determined using a calibration curve obtained with standard proteins, as previously described[5].

**Proteolysis of hTTR**. hTTR samples of (50 μl; 1 mg/ml, 18 μM tetramer) in TBS, containing 5 mM $CaCl_2$ (TBS-$CaCl_2$) were incubated at 37°C in the presence of the selected protease, at a protease:hTTR molar ratio of 1:20. After 24h-reaction, aliquots (10 μg) of each proteolysis mixture

was quenched with $H_2O$/TFA-0.1% (v/v) and then analysed by reducing SDS-PAGE (4-15% acrylamide and Coomassie staining) and RP-HPLC using a (4.6 x 300 mm) Zorbax 300SB-C18 column (Agilent Technologies), eluted (0.8 ml/min) with a linear acetonitrile-0.1% TFA gradient. The chemical identity of the proteolytic fragments was established by LC-MS analysis by loading acid quenched aliquots (35 μg) of the reaction mixture, after 10-h proteolysis, onto a C18 microbore column eluted (50 μl/min) with a linear aqueous acetonitrile-0.1% (v/v) formic acid gradient. For the kinetic analyses, hTTR (100 μl, 1 mg/ml) was incubated with subtilisin (1: 20 molar ratio at 37°C); aliquots (20 μl) were taken, acid quenched with 0.1% (v/v) aqueous TFA (80 μl), frozen at -20°C and then analysed by reducing SDS-PAGE and RP-HPLC as above. Quantitative determination of intact hTTR and hTTR(59-127) fragment was performed by densitometric analysis of the electrophoretic gel bands, using the Geliance-600 Chem-Imaging system (Perkin-Elmer, Waltham, MA, USA), or by integrating the area under the chromatographic peaks, using molar absorptivity values at 226 nm of 10.2 and 6.8 $cm^2 \cdot mg^{-1}$ for intact and fragmented protein, respectively. Kinetic data of hTTR degradation were interpolated with equation 1, describing a pseudo-first order reaction:

$$[hTTR]_t = [hTTR]_0 \cdot e^{-kt} \qquad \text{eq. 1}$$

where $[hTTR]_0$ and $[hTTR]_t$ is the hTTR concentration at the beginning of the reaction and after time *t*, respectively, and *k* is the observed cumulative kinetic constant of hydrolysis[6]. For preparative purposes, 1-ml aliquots of purified hTTR (18 μM) were incubated for 10 h with subtilisin (0.9 μM). The reaction was stopped with 4% (v/v) aqueous TFA (25 μl) and hTTR(59-127) purified by RP-HPLC on a (1 x 25 cm) semi-preparative (Grace-Vydac) C18 column eluted (1.5 ml/min) with a linear acetonitrile-0.078% TFA gradient from 10 to 55% in 45 min. hTTR(59-127), eluting with the major chromatographic peak, was collected, immediately frozen at -20°C and lyophilised in a Savant SpeedVac concentrator (Thermo-Fischer Scientific, Waltham, MA, USA). After lyophilisation, the fragment was dissolved in 10 mM phosphate buffer, pH 7.4, and tested for amyloid formation.

Proteolysis of hTTR in human plasma was conducted by incubating plasma samples (800 μl) with increasing concentrations of subtilisin at 37°C for 16 h, under static or dynamic conditions (500 rpm) using a Thermomixer Compact (Eppendorf-AG, Hamburg, Germany). Human plasma was obtained from the Institutional Blood Bank of the University Hospital of Padua. Residual intact hTTR and the newly generated hTTR(59-127) were recovered by the phenol precipitation method (see above), properly downscaled to small volumes of human plasma. Intact hTTR and hTTR(59-

127) were identified by reducing SDS-PAGE (4-14% acrylamide, Coomassie staining) and UPLC-MS. Protein bands were excised from the acrylamide gel and subjected to *in situ* tryptic digestion and peptide mass fingerprint analysis[2]. For UPLC-MS analysis, lyophilized samples were dissolved in 5%-acetonitrile aqueous solution (45 ul), containing 0.1% formic acid, and loaded onto a (2.1 x 150 mm, 2.7 µm) AdvanceBio PeptideMap Agilent (Santa Clara, CA, USA) column, heated at 30°C and eluted (0.2 ml/min) with a linear acetonitrile-0.1% formic acid gradient from 5% to 70% in 30 min. S-Cys-hTTR isoform and hTTR(59-127) fragment were identified by searching the multiple-charged species of highest intensity, i.e. 817.47 m/z for hTTR and 970.49 m/z for hTTR(59-127), in the Total Ion Current (TIC) trace. The extracted ion chromatograms (XICs) were obtained from the TIC plots using the DisplayMass tool, as implemented in the MassLynx software, and setting the experimental error at 6-ppm and the intensity threshold at $10^3$ counts.

**HDX-MS: global exchange analysis.** The kinetics of deuterium incorporation into intact proteins was determined by measuring the deuteration level (%D) at increasing incubation times with $D_2O$ at 25°C, according to equation 2[2,3]:

$$\%D = (m_t - m_0)/(m_{100} - m_0) \qquad \text{eq. 2}$$

where $m_t$ is the mass of the protein after labelling time $t$, $m_0$ and $m_{100}$ are the mass of the protein before or after deuteration, taking into account back-exchange effects. The value of $m_t$ was determined by first adding 7.5 µl of 10 mM sodium phosphate buffer, pH 7.4, 0.15 M NaCl (PBS) to a purified protein solution (7.5 µl, 36 µM monomer) in the same buffer. The resulting solution (15 µl) was then diluted 1:10 (v/v) with $D_2O$ to a final volume of 150 µl. When $m_{100}$ was being determined, 7.5 µl of PBS containing 8 M Gdn-HCl, were added to the protein solution (7.5 µl, 36 µM monomer). To maximise HDX labelling, the denatured protein was heated at 100°C for 5 min. At time points, aliquots (20 µl) of the labelling solution (150 µl, 90% $D_2O$) were acid quenched at 0°C with 35 mM HCl (20 µl) and immediately frozen in liquid nitrogen for subsequent LC-MS analysis. Each aliquot was thawed for 20 sec at room temperature and injected on a (1 x 50 mm) microbore C4 column (Grace-Vydac). The column was eluted (100 µl/min) with a linear acetonitrile-0.1% (v/v) formic acid gradient from 10 to 50% in 8 min and analysed as detailed above. To minimise back-exchange effects, the eluents, the injection valve, and the column were thermostated at 0°C in water ice bath. MaxEnt software, as implemented in MassLynx suite, was used to deconvolute *m/z* spectra and obtain the $m_t$ values.

**HDX-MS: local exchange analysis.** Samples were prepared by incubating hTTR (15 μl, 60 μM) with 285 μl of $D_2O$ (95% in PBS, pH 7.1) at 22°C. Aliquots (30 μl) were withdrawn and added to a pre-chilled (0°C) quenching solution (30 μl, 200 mM sodium phosphate, pH 2.4, 1.5 M Gdn-HCl) and immediately frozen in liquid nitrogen and stored at -80°C for LC-MS analysis. For on-line pepsin digestion, each aliquot was quickly thawed to 0 °C and injected onto a (2.1 × 20 mm) Poroszyme Immobilized Pepsin Cartridge (Applied Biosystems, Foster City, CA, USA) thermostated at 20°C and equilibrated with 100% buffer A (0.23% formic acid in 2% aqueous acetonitrile) at a flow rate of 90 μl/min. The peptides eluting from the Pepsin Cartridge were on-line trapped, concentrated, and desalted on a (1.7 μm, 2.1 × 5 mm) C18 VanGuard BEH Precolumn, connected on-line *via* a switching valve to a (1.7 μm, 2.1 × 50 mm) Acquity UPLC BEH C18 column (Waters). After 3-min desalting with buffer A, the precolumn and RP column were equilibrated with 5% aqueous acetonitrile-0.1% formic acid and eluted (90 μl/min) with a linear acetonitrile-0.1% formic acid gradient from 5% to 60% in 10 min.

Mass spectra were acquired in the resolution mode (m/z 50–2000) on a Waters Xevo G2S, equipped with a standard electrospray ionization source. Leu-enkephalin (Waters) was continuously infused as the reference lock mass. Each peptide was identified in the $MS^E$ mode, using argon as collision gas. Only those fragments matching the following criteria were considered: i) a 0.3-min retention time window in the chromatographic separation; ii) a mass accuracy of the precursor ion < 6 ppm; iii) a mass accuracy of the product ions < 16 ppm; iv) a R value > 0.3 for each fragment, where R is the ratio of the number of product ions *per* amino acid residue. Peptides containing less than 5 or more than 30 amino acids were not considered, due to increased ambiguity and poor sequence localization. Data were processed using the BioPharmaLynx suite (Waters) and each fragmentation spectrum was manually inspected to confirm the reliability of the automated assignment, according to the criteria reported above. The HDExaminer vs. 2.5 software (Sierra Analytics, Modesto, CA, USA) was used to calculate %D values for the selected fragments and then to generate the heatmap of %D. Notably, the data for the first two residues in peptic fragments were not included in %D calculations, as they undergo very fast back-exchange[2]. All HDX measurements were conducted in triplicate with the error as the standard deviation.

**UV-Vis absorption spectroscopy.** Protein concentration was determined by measuring the absorption of protein solutions at 280 nm on a Jasco (Tokyo, Japan) V-630 double beam spectrophotometer, using an absorptivity ($\varepsilon_{0.1\%}$) value of 1.35 $mg^{-1}\cdot cm^2$ for intact hTTR and 1.67 $mg^{-1}\cdot cm^2$ for hTTR(59-127).

**Turbidimetry** The time-course aggregation of hTTR(59-127) was monitored by recording the turbidimetric signal[6] of the proteolysis mixture of hTTR (18 μM as tetramer) with subtilisin (0.9 μM) at 37°C in TBS-$CaCl_2$. At time points, aliquots (50 μl) of the reaction mixture were taken, frozen at -20°C and used for subsequent analyses. After thawing at room temperature (r.t.), each aliquot was diluted (1:10 v/v) with TBS-$CaCl_2$ and the absorbance ratio ($Abs_{260}/Abs_{280}$) in the UV spectrum was measured.

**Circular dichroism.** Measurements were carried out on a Jasco J-1500 spectropolarimeter, equipped with a Peltier temperature control system, as detailed elesewhere[7]. CD spectra were recorded at a scan-speed of 10 nm/min, with a response time of 16 sec, and resulted from the average of four accumulations after baseline subtraction. CD signal was expressed as the mean residue ellipticity $[\theta] = \theta_{obs} \cdot MRW/(10 \cdot l \cdot c)$, where $\theta_{obs}$ is the observed signal in degrees, MRW is the protein mean residue weight, l is the cuvette pathlength in cm, and c is the protein concentration in g/ml.

**Fluorescence and ThT binding assay.** Fluorescence spectra were recorded on a Jasco FP-6500 spectrofluorimeter, equipped with a Peltier temperature control system, at a scan speed of 200 nm/min and excitation/emission slits of 3/3 nm. The final spectra were the average of four accumulations, after baseline subtraction, while photobleaching of Trp-residues was found < 1%. Amyloid fibril formation was assessed by ThT fluorescence binding assay[8]. hTTR (18 μM as tetramer) was incubated at 37°C with subtilisin (0.9 μM) in TBS-$CaCl_2$. At time points, aliquots (50 μl) were taken and stored at -20°C. After thawing at r.t., aliquots were diluted (1:10 v/v) with TBS-$CaCl_2$. To 400 μl of the diluted hTTR solution 100 μl of a ThT stock solution (100 μM) in the same buffer were added. After 1-h incubation with ThT at 25°C, samples were excited at 450 nm and the emission intensity was recorded at 482nm, with excitation/emission slits of 3/10 nm. For hTTR(59-127), the purified fragment was incubated (0.23 mg/ml) over time in TBS at 37°C. At time points, aliquots (108 μl) were taken, diluted to 400 μl with TBS and added with 100 μl of a ThT stock solution (100 μM). After 1-h incubation with ThT, fluorescence spectra were recorded as detailed above.

**DLS analysis**. DLS measurements were performed in TBS on a Zetasizer Nano-S instrument (Malvern Instruments, UK), using a laser source at 633 nm and at fixed angle (173°) from the incident light. Disposable polystyrene cuvettes (1-cm pathlength, 100μl) (Hellma, Switzerland) were used and each measurement consisted of a single run (15s). The refractive index (n) and

viscosity (η) of the protein solutions were taken as 1.450 and 0.887cP, respectively. DLS measurements yield the values of $R_h$ and %PD, where $R_H$ is the hydrodynamic radius, i.e. the radius of a hard sphere that diffuses at the same rate as that of the molecule considered, and %PD is the polydispersity index, i.e. the width of the particle size distribution of a protein in a sample[9]. Scattering data were analyzed with the Nano-6.20 software and expressed as % mass or % intensity size distribution. The reliability of DLS measurements was estimated from the value of the correlation coefficient (> 0.5), extracted from the autocorrelation curve.

**TEM measurements.** TEM analyses of the hTTR/subtilisin proteolysis mixture and of purified hTTR(59-127) fragment were carried out after 72-h reaction/incubation in TBS-$CaCl_2$ or TBS alone at pH 7.4. As a positive control, TEM micrographs of acid-induced hTTR fibrils (generated after 72-h incubation of the intact protein in 0.1 M potassium acetate buffer, pH 4.4) were also taken. A drop of the sample solution was placed on a Butvar-coated copper grid (400-square mesh) (TAAB-Laboratories Equipment Ltd, Berks, UK), air dried and negatively stained with 1.0% (w/v) uranyl acetate solution. TEM micrographs were taken on a Tecnai G2 12 Twin instrument (FEI Company, OR, USA) using an excitation voltage of 100 kV.

**Derivatisation of subtilisin with FITC and PMSF**. A FITC solution in DMSO (220 µl, 51 mM) was reacted with subtilisin (600 µl, 73 µM) in 0.1 M $Na_2CO_3$ buffer, pH 9.0, for 3 h in the dark at 25°C, to yield FTC-subtilisin. FTC-subtilisin (0.5 mM) was then irreversibly inhibited at the active-site after incubation with PMSF (2 mM) in 0.1 M $Na_2CO_3$, pH 9.0, for 2 hours at 25°C, to yield PMS-FTC-subtilisin. The reaction mixtures were desalted on a HiTrap G-25 fast-flow SEC column (5 ml) (GE-Healthcare), eluted (1.0 ml/min) with PBS. The chemical identity of subtilisin derivatives was established by high-resolution MS, yielding mass values compatible with the incorporation of a single FTC- or PMS-moiety. The residual activity of PMS-FTC-subtilisin was determined on the chromogenic substrate Azocoll, according to the manufacturer's procedure (see the legend to Supplementary Figure 10). For Transwell assays (see below), labelled subtilisin samples were desalted on a Zeba-Spin column (0.5 ml) (Thermo-Fischer Scientific, Waltham, MA, USA) in 10 mM HEPES buffer, pH 7.4, 10 mM glucose.

**Caco-2 cells permeability assay**. Permeability of the intestinal epithelium to subtilisin was estimated *in vitro* by challenging a monolayer of Caco-2 (human epithelial colorectal adenocarcinoma) cells with the fluorescein-labelled enzyme, both in its active (FTC-subtilisin) and inactivated (PMS-FTC-subtilisin) form. Caco-2 cells (American Type Culture Collection, Manassas, VA, USA) were grown in high-glucose Dulbecco's modified Eagle's media (DMEM)

containing 20% (v/v) fetal calf serum, 2% L-glutamine and 1% (w/v) penicillin and streptomycin. Cells were trypsinized and seeded in the upper chamber of Transwell polyester membrane cell culture inserts (1.0 $cm^2$ growth surface area, 0.4 μm pore size) in a 24-wells sterile plate. Medium was replaced in upper and lower chambers every 24 hours. After 14 days, the formation of confluent cellular monolayers with well-developed tight junctions was confirmed by Transepithelial Electrical Resistance (TEER) measurements using a Millicell-ERS meter (Millipore Corporation, Bedford, MA, USA) connected to chopstick electrodes. The integrity of Caco-2 monolayers was verified by TEER measurements and only values of 500–700 ohm/$cm^2$ for three consecutive days were considered acceptable. FTC-subtilisin or PMS-FTC-subtilisin in DMEM (0.2 μM, 400 μl) were added to the apical chamber of the Transwell inserts. Aliquots (50 μl) were collected from the basolateral compartment at 2-h intervals, placed in a 96-wells plate, and excited at 488 nm, while the emission intensity was measured at 535 nm on a Victor X2 (Perkin-Elmer) plate reader. Subtilisin translocation was quantified using a calibration curve obtained with known concentrations of labelled enzyme. Blank permeability experiments were performed with DMEM alone.

## Supplementary References


1. Poulsen, K., Bahl, J. M., Tanassi, J. T., Simonsen, A. H. & Heegaard, N. H. Characterization and stability of transthyretin isoforms in cerebrospinal fluid examined by immunoprecipitation and high-resolution mass spectrometry of intact protein. *Methods* **56**, 284–292 (2012).
2. Sowole, M. A. & Konermann, L. Effects of protein-ligand interactions on hydrogen/deuterium exchange kinetics: canonical and noncanonical scenarios. *Anal. Chem.* **86**, 6715–6722 (2014).
3. Masson, G. R. *et al.* Recommendations for performing, interpreting and reporting hydrogen deuterium exchange mass spectrometry (HDX-MS) experiments. *Nat. Methods* **16**, 595–602 (2019).
4. Colon, W. & Kelly, J. W. Partial denaturation of transthyretin is sufficient for amyloid fibril formation in vitro. *Biochemistry* **31**, 8654–8660 (1992).
5. Sokolov, A. V. *et al.* Thrombin inhibits the anti-myeloperoxidase and ferroxidase functions of ceruloplasmin: relevance in rheumatoid arthritis. *Free Radic. Biol. Med.* **86**, 279–294 (2015).
6. Pontarollo, G. *et al.* Non-canonical proteolytic activation of human prothrombin by subtilisin from Bacillus subtilis may shift the procoagulant–anticoagulant equilibrium toward thrombosis. *J. Biol. Chem.* **292**, 15161–15179 (2017).
7. De Filippis, V., Colombo, G., Russo, I., Spadari, B. & Fontana, A. Probing the hirudin-thrombin interaction by incorporation of noncoded amino acids and molecular dynamics simulation. *Biochemistry* **41**, 13556–69 (2002).
8. Nilsson, M. R. Techniques to study amyloid fibril formation in vitro. *Methods* **34**, 151–160 (2004).
9. Acquasaliente, L. *et al.* Molecular mapping of alpha-thrombin (alphaT)/beta2-glycoprotein I (beta2GpI) interaction reveals how beta2GpI affects alphaT functions. *Biochem. J.* **473**, 4629–4650 (2016).
